# Comparing the Clinical Outcomes between Drug Eluting Stents and Bare Metal Stents in Patients with Insulin-Treated Type 2 Diabetes Mellitus: A Systematic Review and Meta-Analysis of 10 Randomized Controlled Trials

**DOI:** 10.1371/journal.pone.0154064

**Published:** 2016-04-25

**Authors:** Pravesh Kumar Bundhun, Akash Bhurtu, Mohammad Zafooruddin Sani Soogund, Man-Yun Long

**Affiliations:** 1 Institute of Cardiovascular Diseases, the First Affiliated Hospital of Guangxi Medical University, Nanning, Guangxi, 530021, P. R. China; 2 Guangxi Medical University, Nanning, Guangxi, 530021, P. R. China; Harefield Hospital, UNITED KINGDOM

## Abstract

**Background:**

Several studies have shown Drug Eluting Stents (DES) to be better compared to Bare Metal Stents (BMS) in patients with type 2 Diabetes Mellitus (T2DM). Since, the adverse clinical outcomes in patients with Insulin-Treated Type 2 Diabetes Mellitus (ITDM) implanted with DES and BMS have not been previously studied, we aim to compare the clinical outcomes in similar patients with cardiovascular diseases, treated with DES and BMS.

**Methods:**

Randomized Controlled Trials (RCTs) comparing patients treated with DES and BMS were searched from PubMed and EMBASE databases. Outcome data for the patients with ITDM were carefully extracted. Major Adverse Cardiac Events (MACEs), mortality, Target Vessel Revascularization (TVR), Target Lesion Revascularization (TLR), Myocardial Infarction (MI) and Stent Thrombosis (ST) were considered as the clinical endpoints for this analysis. Odds ratios (OR) with 95% confidence intervals (CIs) were calculated and the pooled analyses were performed with RevMan 5.3 software.

**Results:**

Ten RCTs consisting of 830 patients with ITDM (477 patients in the DES group and 353 patients in the BMS group) from a total number of 9,141 patients were included in this analysis. During a follow-up period from one month to one year, MACEs were not increased with the use of DES in these patients with ITDM. At 9 months, MACEs were significantly lower in the DES group with OR: 0.40, 95% CI: 0.23–0.72; P = 0.002 with no increase in mortality. TVR and TLR also favored the DES group with OR: 0.44, 95% CI: 0.22–0.88, P = 0.02 and OR: 0.28, 95% CI: 0.14–0.53; P = 0.0001 respectively at 9 months, and OR: 0.46, 95% CI: 0.23–0.94, P = 0.03 and OR: 0.28, 95% CI: 0.14–0.55; P = 0.0003 respectively at one year. Results for MI, and ST were not statistically significant.

**Conclusion:**

Compared to BMS, DES were associated with a significantly lower rate of repeated revascularization, without any increase in MACEs or mortality in these patients with ITDM during a follow up period of one year. However, due to the very small population size, further studies with a larger number of randomized patients are required to completely solve this issue.

## Introduction

Today, an estimated 171 million people suffer from Diabetes Mellitus (DM) all over the world and the prevalence of this chronic disease is expected to double over the next two decades [1]. DM is associated with a higher rate of repeated revascularization compared to non-DM [2]. Even if studies have shown that type 2 Diabetes Mellitus (T2DM) is independently associated with significantly higher adverse clinical outcomes whether with Drug Eluting Stents (DES) or Bare Metal Stents (BMS) [3], compared to non-T2DM, other studies have shown DES to have been associated with better outcomes compared to BMS in patients with T2DM. For example, the pooled analysis of seven Randomized Controlled Trials (RCTs) by Lijima et al showed that DES significantly improved adverse outcomes compared to BMS, in patients with T2DM who underwent Percutaneous Coronary Intervention (PCI) for Acute Myocardial Infarction (AMI) [4]. However, because of contraindications to the use of DES, BMS is still considered equally important in several subgroups of patients. T2DM can be further divided into Insulin-Treated Diabetes Mellitus (ITDM) (requiring insulin therapy with or without oral hypoglycemic medications as treatment) and non-ITDM (require only diet control and oral hypoglycemic agents as treatment). Since insulin therapy is associated with increased adverse cardiovascular outcomes after PCI [5] and because the adverse clinical outcomes observed with the use of DES and BMS have not been previously studied in patients with ITDM, we aim to solve this issue using data only from RCTs.

## Methods

### Search Strategy

RCTs comparing DES with BMS were searched from Medline and EMBASE by typing the words ‘drug eluting stents, bare metal stents, and/or percutaneous coronary intervention’. Abbreviations (DES, BMS and/or PCI) were also used. To widen the search or simply to be more specific, the word ‘diabetes mellitus’ was also included. No language restriction was applied.

### Inclusion and Exclusion Criteria

**Studies were included if:**

1. They were RCTs.
2. They compared DES with BMS.
3. They included data for patients with ITDM.
4. They reported adverse outcomes as their clinical endpoints.

**Studies were excluded if:**

1. They were non-RCTs for example observational studies, case studies or meta-analyses.
2. They did not include patients with ITDM.
3. Adverse outcomes were not reported among their clinical endpoints.

### Defining outcomes and follow-up period

**The reported outcomes were:**

- Major Adverse Cardiac Events (MACEs)
- Mortality
- Myocardial Infarction (MI)
- Repeated revascularization including Target Vessel Revascularization (TVR) and Target Lesion Revascularization (TLR)
- Stent Thrombosis (ST)

The follow-up period varied from one month to one year. The reported outcomes and their corresponding follow up periods have been listed in Table 1.

**Table 1:** The adverse outcomes reported in the included trials. Abbreviations: MACEs: major adverse cardiac events, MI: myocardial infarction, TLR: target lesion revascularization, TVR: target vessel revascularization, ST: stent thrombosis.

Trials	Reported Outcomes	Follow up period (months)
**SCORPIUS**	Mortality, MI and TLR	1, 8 and 12
**PASEO**	Mortality, MI, TLR, MACEs, ST	12
**DIABETES**	MACEs, revascularization	1 and 9
**HORIZONS-AMI**	Mortality, MACEs, TLR, TVR, ST, MI	12
**PRODIGY**	TVR, TLR, ST	24
**TAXUS VI**	Mortality, MACEs, TVR, TLR, MI	1 and 9
**TAXUS IV**	MACEs, TVR, TLR	12
**SIRIUS**	MACEs, TLR	9
**STONE2005**	Mortality, MACEs, TLR, TVR, MI, ST	1 and 9
**STONE2004**	Mortality, MI, ST, TVR, TLR, MACEs	9

### Data Extraction and Analysis

Three authors (P.K.B, A.B and M.Z.S.S) independently reviewed the data and each of them assessed whether the trials were eligible for this study. The methodological quality of all the trials were also assessed. First of all, outcome data for the patients with ITDM were carefully extracted. Then information regarding the study type, the author names, the year of publications and the patient characteristics as well as the clinical outcomes reported and the follow up periods was systematically extracted. Disagreements raised were discussed among the authors, and then a decision was made. However, if the authors could not reach a consensus, disagreements were resolved by the fourth author (M.Y.L). Risk of bias among the trials was assessed in accordance to the components recommended by the Cochrane Collaboration [6].

### Statistical Analysis

Recommendations of the PRISMA (Preferred Reporting Items for Systematic Reviews and Meta-*Analyses*) statement were considered [7]. Heterogeneity across trials was assessed using the Cochrane Q-statistic (whereby P ≤0.05 was considered statistically significant whereas P > 0.05 was considered statistically insignificant) and also using the I^2^-statistic whereby I^2^ described the percentage of total variation across studies (a low value of I^2^ indicated lower heterogeneity whereas a larger value indicated increased heterogeneity). A fixed effect model was used if I^2^ was <50%. However, if I^2^ was >50%, a random effect model was used. Publication bias, which was also taken into consideration in our study, was visually estimated by assessing the funnel plots. We calculated odds ratios (OR) and 95% confidence intervals (CIs) for categorical variables and the pooled analyses of data from our included studies were performed with RevMan 5.3 software.

### Ethics

Ethical approval was not necessary for Systematic Review and Meta-Analysis.

## Results

### Study selection

**Fig 1:**
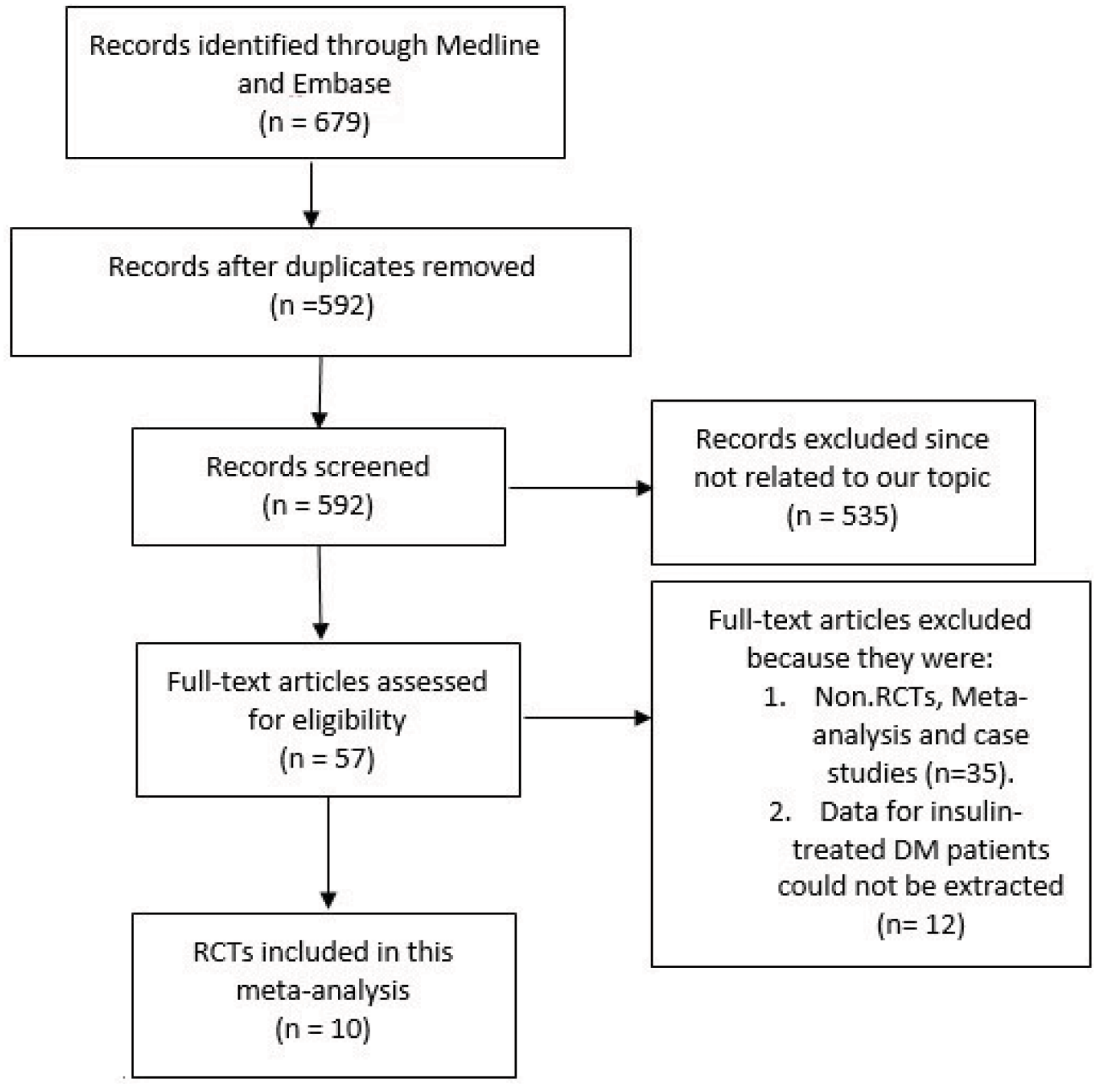
The flow diagram for the study selection.

679 articles were searched from Medline and EMBASE databases. After eliminating the duplicate studies and those studies not related to our topic, 57 full text articles were assessed for eligibility. After another careful screening with strict considering to the inclusion and exclusion criteria of our meta-analysis, 35 more articles were eliminated because they were either: meta-analyses, non-RCTs and case studies. Further 12 articles were eliminated because data for patients with ITDM could not be extracted. Finally, 10 RCTs were selected for this meta-analysis [8–17]. Fig 1 represents the flow chart for the study selection.

A total number of 830 patients with ITDM (including 477 patients from the DES group and 353 patients from the BMS group) were extracted from a total number of 9141 patients (including 5855 patients from the DES group and 3286 patients from the BMS group). Table 2 lists the number of patients with ITDM which were extracted from the DES and BMS groups, as well as the types of DES used in those trials.

**Table 2:** The total number of patients included from each trials. Abbreviations: DES: drug eluting stents, BMS: bare metal stents, PES: paclitaxel eluting stents, SES: sirolimus eluting stents, ZES: zotarolimus eluting stents, EES: everolimus eluting stents, ITDM: insulin-treated diabetes mellitus.

Trials	Type of DES	Total no of patients treated with DES (n)	ITDM patients in the DES group (n)	Total no of patients treated with BMS (n)	ITDM patients in the BMS group (n)
**SCORPIUS**	SES	94	40	96	39
**PASEO**	PES, SES	180	15	90	8
**DIABETES**	SES	80	26	80	27
**HORIZONS-AMI**	PES	2256	98	749	31
**PRODIGY**	ZES, PES, EES	1501	94	502	23
**TAXUS VI**	PES	219	15	227	20
**TAXUS IV**	PES	155	51	163	54
**SIRIUS**	SES	131	38	148	44
**STONE2005**	PES	577	49	579	53
**STONE2004**	PES	662	51	652	54

### Baseline characteristics

The baseline features of these included trials have been summarized in Table 3. On average, more than 60% of the population were males with a mean age above 60 years old. No significant differences have been observed in the baseline features among patients classified under the DES and BMS groups. Further details are listed in Table 3.

**Table 3:** The baseline features reported within the included trials. Abbreviations: DES: drug eluting stents, BMS: bare metal stents.

Trials	Age (years)	Males	Hypertension	Dyslipidemia	Smoker
	*DES/BMS*	*DES/BMS*	*DES/BMS*	*DES/BMS*	*DES/BMS*
**SCORPIUS**	66.0/66.0	66.0/62.0	93.0/94.0	82.0/80.0	20.0/18.0
**PASEO**	62.5/62.0	70.0/71.1	27.3/24.4	-	24.4/26.7
**DIABETES**	65.2/67.9	62.5/62.5	66.3/66.3	61.3/61.3	45.0/50.0
**HORIZONS-AMI**	59.9/59.3	77.0/76.0	51.2/51.9	42.2/41.1	46.3/51.9
**PRODIGY**	68.0/69.0	77.3/74.0	71.0/75.0	56.0/51.0	23.3/25.0
**TAXUS VI**	61.8/63.4	76.3/76.2	57.5/58.1	70.3/73.4	22.5/23.9
**TAXUS IV**	62.6/61.8	59.4/67.5	79.4/82.8	77.0/66.0	-
**STONE2005**	62.9/62.8	70.2/68.7	76.4/73.6	72.3/73.9	21.1/19.9
**STONE2004**	62.8/62.1	71.8/72.4	70.5/69.0	65.0/65.6	23.4/20.1

Percentage (%) was used to represent the baseline characteristics listed in Table 3.

Baseline feature for the SIRIUS trial was not provided in the original manuscript and hence has been omitted in our study.

### Main result of this study

Results of this meta-analysis have been summarized in Table 4.

**Table 4:** Shows the results of this analysis. Abbreviations: OR: odds ratio, MACEs: major adverse cardiac events, MI: myocardial infarction, TVR: target vessel revascularization, TLR: target lesion revascularization, ST: stent thrombosis.

Outcomes	Number of participants (n)	OR with 95% CI	P value	I^2^ (%)
***One month***				
**MACEs**	190	0.70 [0.18–2.74]	0.61	0
**MI**	216	0.86 [0.21–3.55]	0.83	0
***Nine months***				
**MACEs**	377	0.40 [0.23–0.72]	0.002	0
**Mortality**	321	1.38 [0.30–6.32]	0.67	0
**MI**	321	1.24 [0.44–3.51]	0.68	0
**TVR**	324	0.44 [0.22–0.88]	0.02	0
**TLR**	456	0.28 [0.14–0.53]	0.0001	0
***12 months***				
**MACEs**	374	0.65 [0.36–1.19]	0.16	0
**Mortality**	336	0.74 [0.22–2.42]	0.62	0
**MI**	453	0.50 [0.22–1.13]	0.10	0
**TVR**	351	0.46 [0.23–0.94]	0.03	0
**TLR**	453	0.28 [0.14–0.55]	0.0003	0
**ST**	269	0.71 [0.13–3.72]	0.68	0

#### Adverse clinical outcomes at one month follow up

At one month, 130 patients from the DES group and 139 patients from the BMS group, with a total number of 269 patients classified under the ITDM subgroup were analyzed.

**Fig 2:**
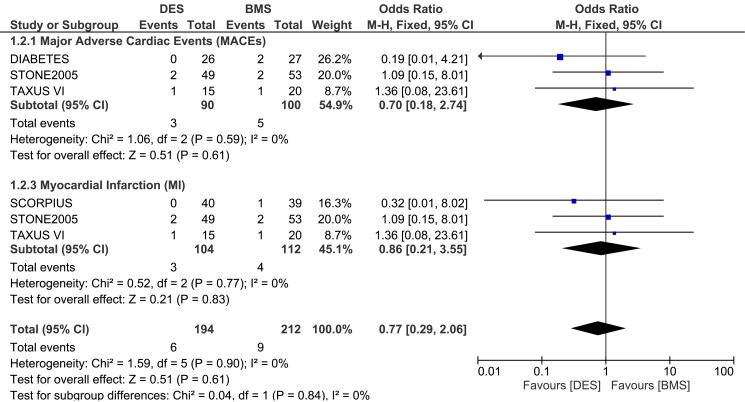
Forest plot comparing the adverse clinical outcomes between DES and BMS in patients with insulin-treated diabetes mellitus during a one month follow up.

The pooled analysis showed that MACEs were not increased with the use of DES in these patients with ITDM, OR: 0.70, 95% CI: 0.18–2.74; P = 0.61. Results for MI was also not statistically significant between DES and BMS with OR: 0.86, 95% CI: 0.21–3.55; P = 0.83. This result has been illustrated in Fig 2.

#### Adverse clinical outcomes at nine months follow up

**Fig 3:**
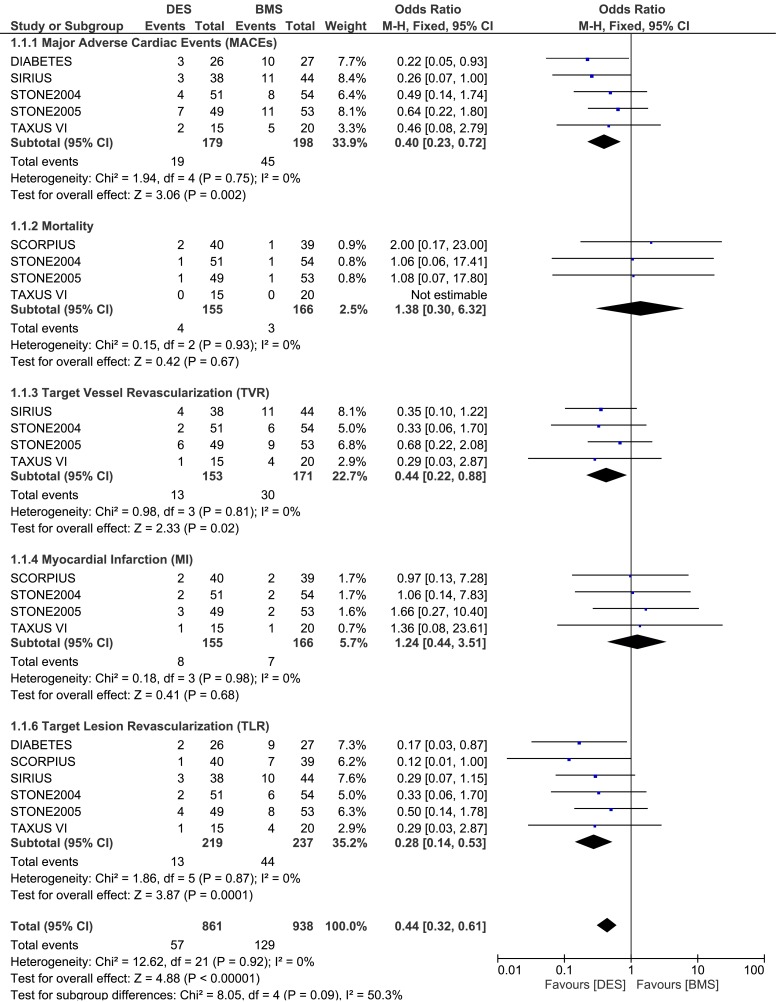
Forest plot comparing the adverse clinical outcomes between DES and BMS in patients with insulin-treated diabetes mellitus during a follow up period of nine months.

During a follow up of 9 months, whereby 219 patients from the DES group and 237 patients from the BMS group were analyzed, MACEs were significantly lower in the DES group with OR: 0.40, 95% CI: 0.23–0.72, P = 0.002. Revascularization including TVR and TLR were also significantly lower in the DES group with OR: 0.44, 95% CI: 0.22–0.88; P = 0.02 and OR: 0.28, 95% CI: 0.14–0.53, P = 0.0001 respectively. However, results for mortality and MI were not statistically significant. Result for the 9 months follow up period has been illustrated in Fig 3.

#### Adverse clinical outcomes at one year follow up

**Fig 4:**
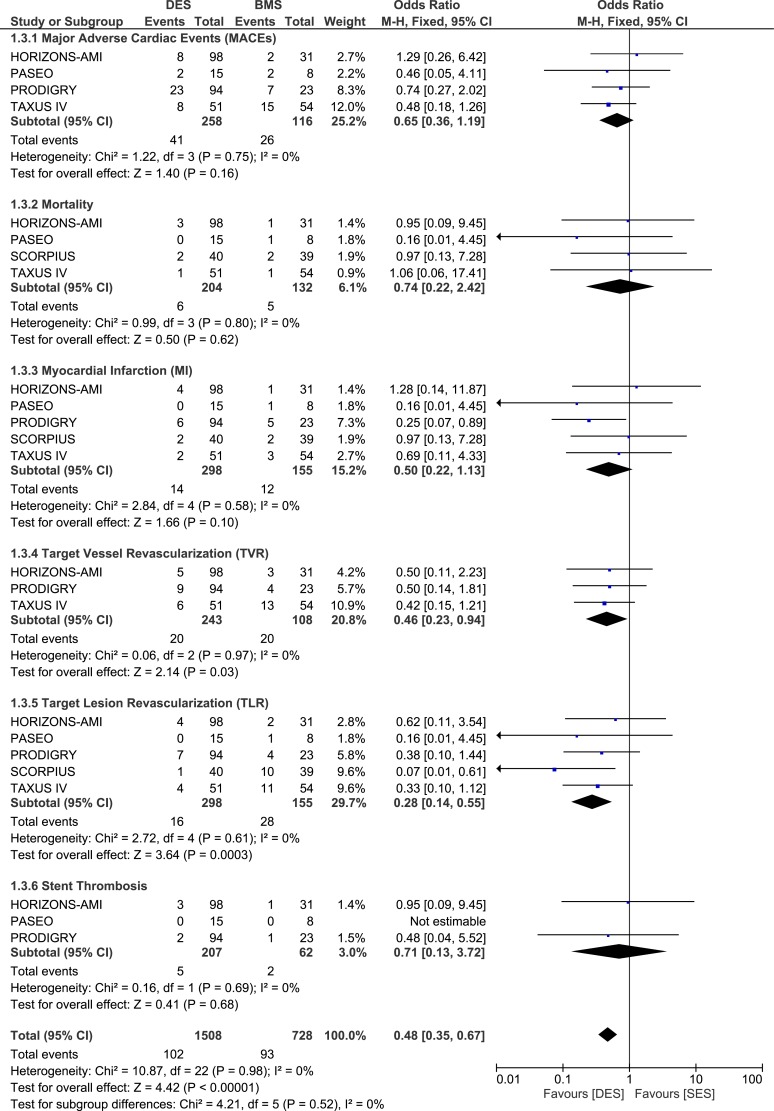
Forest plot comparing the adverse clinical outcomes between DES and BMS in patients with insulin-treated diabetes mellitus during a one year follow up.

During the one year follow up, whereby 298 patients with ITDM in the DES group and 155 patients with ITDM in the BMS group were analyzed, MACEs did not increase with the use of DES with OR: 0.65, 95% CI: 0.36–1.19; P = 0.16. Similarly, mortality rate did not increase. TVR and TLR were still significantly reduced in the DES group with OR: 0.46, 95% CI: 0.23–0.94; P = 0.03 and OR: 0.28, 95% CI: 0.14–0.55, P = 0.0003 respectively. MI was lower in the DES group but the result was not statistically significant. No increase in ST was noted. These results have been illustrated in Fig 4.

**Fig 5:**
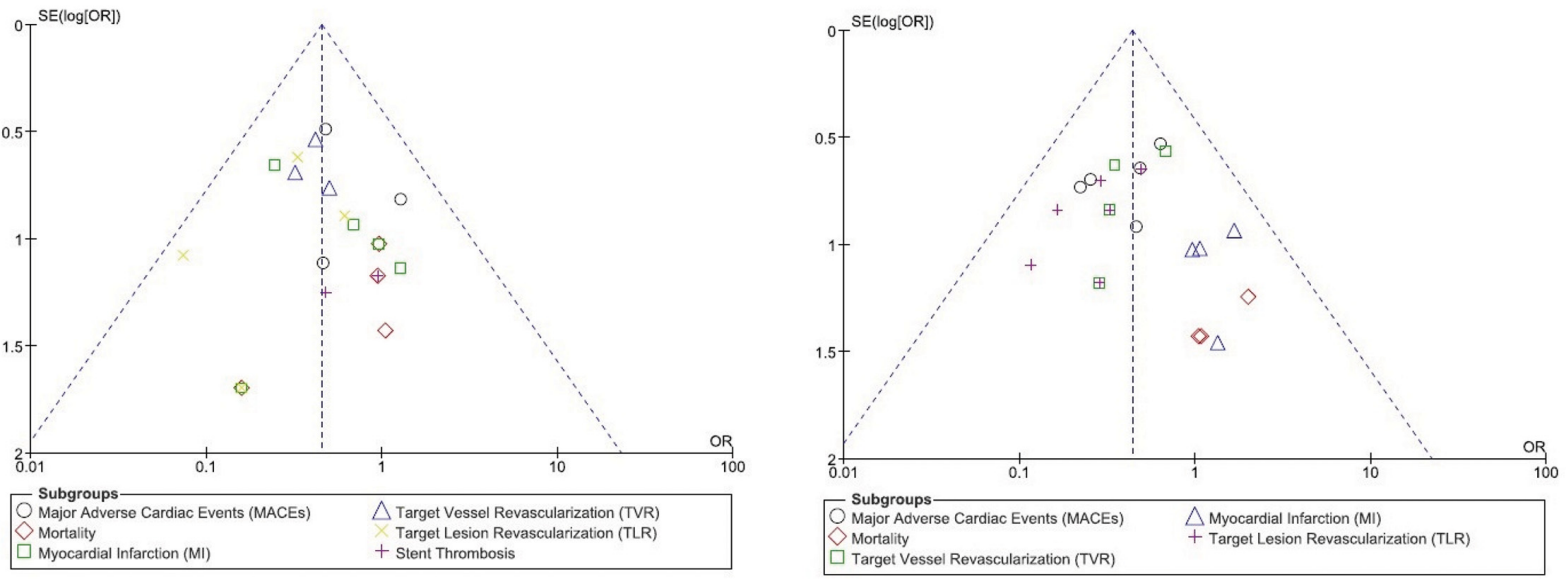
(A and B): funnel plots representing the sensitivity analysis.

For all of the above analyses, sensitivity analyses yielded consistent results. Based on a visual inspection of the funnel plots, there has been no evidence of publication bias observed among the included trials that assessed all clinical endpoints in this study. The funnel plots have been illustrated in (Fig 5A and 5B).

## Discussion

Nowadays DES are the most widely used stents in PCI centers. Several studies have shown DES to be better compared to BMS. Patients with T2DM have higher risks of MACEs and repeated revascularization after PCI, and DES have shown to be beneficial to this particular subgroup of patients as well. T2DM can further be classified into ITDM and non-ITDM. Because insulin therapy is expected to be associated with significantly higher cardiovascular outcomes compared to NITDM after PCI [5] and since DES and BMS have not been previously compared in patients with ITDM, we aim to compare the adverse outcomes between DES and BMS in similar patients.

Our results showed that during a follow up period from one month to one year, MACEs and mortality did not increase with the use of DES in these patients with ITDM. TVR and TLR were significantly lower in the DES group indicating that DES are associated with lower repeated revascularization rates compared to BMS. Results for MI and ST were not statistically significant in our study.

Normally, after coronary revascularization, patients with T2DM have an increased risk of Target Vessel Failure (TVF) and repeated revascularization due to a faster restenosis rate compared to patients without DM [18]. Several mechanisms have been suggested to increase the risk of plaque formation and aggravate coronary artery disease [19–21]. Hyperglycemia can result in altered inflammatory pathways, resistance to insulin, and altered free fatty acid metabolism thus causing endothelial dysfunction, increased platelet production, monocyte activation, foam cell transformation and altered smooth muscle cell migration. The increased production of pro-inflammatory cytokines such as tumor necrosis factor and interleukins 6 in these patients with T2DM could contribute to thrombogenesis. Whether patients with T2DM are treated with insulin or not, all patients are at risk of repeated revascularization but however, studies have shown a higher risk of repeated revascularization among patients within the ITDM group indicating that insulin therapy could partly be responsible or could act as an adjuvant thus contributing to such outcomes.

However, compared to BMS, DES are associated with a decreased rate of restenosis and repeated revascularization whether in patients with DM or non-DM [22]. Our results showed DES to significantly reduce the rate of TVR and TLR in these patients with ITDM but however, revascularization rate is expected to be higher in those patients with T2DM who require insulin therapy compared to those who do not require insulin as treatment. Patients with ITDM have the highest rate of restenosis regardless of stent types. Further studies are needed to clarify on the exact reasons for such a result and its association with insulin therapy.

The meta-analysis published by Patti et al comparing DES with BMS in patients with T2DM using data from nine trials, showed patients receiving DES to have lower rate of in-stent restenosis and TLR compared to those patients treated with BMS [23]. A decrease in in-stent restenosis was clearly observed in both, the insulin-treated and non-insulin treated subgroups. DES also significantly reduced the rate of MI without increasing mortality or ST in these patients with T2DM.

Similar to our meta-analysis, the pooled analysis by Lijima et al comparing DES and BMS in patients with T2DM suffering from AMI also showed DES to significantly reduce the revascularization rate without increasing the mortality rate or the rate of MI [4]. Another result from the PRISON II study showed that after three years, TLR was significantly reduced from 27% in the BMS group compared to only 7% in the DES group. Reported TVR was 30% in the BMS group whereas it was only 11% in the DES group [24]. In their study, MACEs were also significantly reduced in the DES group whereby only 10% were observed compared to 34% in the BMS group. However, similar to our result, the rates of mortality, MI and ST were not significant increased between these two groups. Their study compared DES and BMS in patients with total coronary occlusion and had a longer follow up period. However, our study compared DES and BMS in patients with ITDM having a shorter follow up period compared to their study.

Another result from the same trial showed that during a follow up period between 6 months and 1 year, TVR and TLR were significantly lower in the DES group [25]. However, similar to our study, the result for MACEs was not statistically significant. This PRISON II study included only 4% of patients with ITDM in the BMS group. According to two different follow up periods from the same trial, MACEs were still not increased with the use of DES and repeated revascularization was still significantly lower with the use of DES.

The meta-analysis by Kirtane et al comparing the safety and effectiveness of DES and BMS showed a significantly lower revascularization rate associated with the use of DES [26]. However, a similar rate of mortality or MI were observed in the general population analyzed. Moreover, the meta-analysis conducted by Kastrati et al comparing SES and BMS in the general population (involving 14 trials) showed that during a long-term follow up period, SES did not have a significant effect on the overall mortality rate or MI. ST was indifferent to BMS [27]. However, the revascularization rate was significantly reduced with the use of this type of DES. The study by Mauri et al comparing ZES with BMS and the study published by Caixeta et al also showed similar results with our study [28–29].

In addition, the study by Uthamalingum et al comparing the outcomes in octogenarians treated with either DES or BMS showed that at one year, BMS had an increased risk of MACEs compared to DES [30]. The result for death was not statistically significant. Their study included 21.6% of patients with T2DM in the BMS group and 27.5% of patients with T2DM in the DES group. However, the number of patients treated with insulin was unknown.

Our study did not show any significant difference in ST between DES and BMS in these patients with ITDM. Nevertheless, the collaborative network meta-analysis comparing DES and BMS in patients with and without T2DM showed a significant difference in ST between SES and BMS [31]. But Bangalore et al found that SES were not superior to BMS. As a direct corollary, Bangalore et al carried out a large-scale meta-analysis that included 10 714 patients with T2DM, which compared BMS with different types of DES [18]. As this is the most recent study, it is likely that these results are the most reliable.

Another meta-analysis comparing DES and BMS in unprotected left coronary artery stenosis showed results which differed partly from our meta-analysis [32]. Results from that meta-analysis showed a significantly lower revascularization and MACEs during a follow up period of less than one year, an additional significantly lower rate of death at 2 years, and an another addition of significantly lower MI after 3 years in these patients. However, the study included only 85 patients with ITDM in the DES group and 63 patients with ITDM in the BMS group among the 8861 patients with T2DM analyzed.

A comprehensive network meta-analysis by Palmerini et al demonstrated DES to be superior to BMS after a median follow up period of 3.8 years [33]. In this study, cobalt-chromium everolimus eluting stents (EES) were associated with a significantly lower rate of MI, definite ST and death compared to BMS. Revascularization was also significantly lower in the DES group. However, our study comparing DES and BMS in patients with ITDM, did not show any significant decrease in death or MI in the DES group. Our follow up period was also not longer than one year.

DES have proved to be beneficial in T2DM (including both ITDM and NITDM patients). However, controversies still exist about the efficacy of the different types of DES in patients with T2DM. It is now clear than DES are the preferred stents in patients with T2DM including both ITDM and NITDM, but questions are raised about the different types of DES, which have different levels of benefits among these patients with T2DM. For example, when PES, SES and BMS were compared in 3852 patients with T2DM, SES showed to be more beneficial than PES for having a longer event free period and a lower ST at 1 year in these patients with T2DM [30]. Moreover, studies showed EES to be associated with decreased neointima formation, lumen loss and vessel narrowing in patients with T2DM when compared to PES [34]. When EES were compared to SES, similar safety outcomes have been observed. However, EES were associated with a decreased ST and restenosis rate compared to SES [35]. Therefore, not only choosing DES instead of BMS is important, but selecting the correct and most suitable types of DES should also be considered.

Even though BMS were widely used before, they are not commonly preferable to DES. However, even if DES are widely used in this new era, advance research shows the development of carbo and bioresorbable stents to overcome some of the drawbacks of DES. Only future studies can show these differences.

### Limitations

This study also has several limitations. First of all, due to the very small number of patients with ITDM used in this study, this analysis may not generate the required or expected results. Secondly, several trials did not report all-cause mortality among their clinical outcomes. Those studies included: STONE2004, STONE2005 and TAXUS IV and VI. In those studies, cardiac death was considered. Moreover, when comparing DES with BMS during a 9 months follow up period, one study had a follow up period of 8 months and has been included in the comparison. Also, another study had a follow up period of 2 years and because of a limited number of studies with a follow up above one year, we included this study in the one year follow up category for the comparison of outcomes. Trials analyzed for the 9 months follow up included: DIABETES, SIRUS, STONE2004, STONE2005, SCORPIUS and TAXUS VI whereas trials analyzed for the one year follow up included: HORIZONS-AMI, PASEO, PRODIGRY, TAXUS IV and SCORPIUS.

## Conclusion

Compared to BMS, DES were associated with a significantly lower rate of repeated revascularization, without any increase in MACEs or mortality in these patients with ITDM. However, due to the very small population size, further research with a larger number of randomized patients and with a longer follow up period are required to generate a more reliable result.

## Supporting Information

S1 Reference List (DOCX)

S1 Table Checklist (DOC)

S1 Flow Diagram (DOC)

## Abbreviations

T2DM: type 2 diabetes mellitus
ITDM: insulin-treated diabetes mellitus
DES: drug eluting stents
BMS: bare metal stents
MACEs: major adverse cardiac events
PCI: percutaneous coronary intervention
