# Supplementary material for: Comparing the Clinical Outcomes between Drug Eluting Stents and Bare Metal Stents in Patients with Insulin-Treated Type 2 Diabetes Mellitus: A Systematic Review and Meta-Analysis of 10 Randomized Controlled Trials
Source: PLoS One. 2016 Apr 25;11(4):e0154064. doi: 10.1371/journal.pone.0154064 (PMC4844102; doi:10.1371/journal.pone.0154064)
Supplement: S1 Reference List — (DOCX) [file pone.0154064.s001.docx]

**35 excluded articles from our search strategy**

**25 articles were excluded since they were Non-Randomized Controlled Trials:**

# [Simsek C](http://www.ncbi.nlm.nih.gov/pubmed/?term=Simsek%20C%5BAuthor%5D&cauthor=true&cauthor_uid=20965464)^1^, [Magro M](http://www.ncbi.nlm.nih.gov/pubmed/?term=Magro%20M%5BAuthor%5D&cauthor=true&cauthor_uid=20965464), [Boersma E](http://www.ncbi.nlm.nih.gov/pubmed/?term=Boersma%20E%5BAuthor%5D&cauthor=true&cauthor_uid=20965464), [Onuma Y](http://www.ncbi.nlm.nih.gov/pubmed/?term=Onuma%20Y%5BAuthor%5D&cauthor=true&cauthor_uid=20965464), [Nauta ST](http://www.ncbi.nlm.nih.gov/pubmed/?term=Nauta%20ST%5BAuthor%5D&cauthor=true&cauthor_uid=20965464), [Gaspersz MP](http://www.ncbi.nlm.nih.gov/pubmed/?term=Gaspersz%20MP%5BAuthor%5D&cauthor=true&cauthor_uid=20965464), [van der Giessen WJ](http://www.ncbi.nlm.nih.gov/pubmed/?term=van%20der%20Giessen%20WJ%5BAuthor%5D&cauthor=true&cauthor_uid=20965464), [van Domburg RT](http://www.ncbi.nlm.nih.gov/pubmed/?term=van%20Domburg%20RT%5BAuthor%5D&cauthor=true&cauthor_uid=20965464), [Serruys PW](http://www.ncbi.nlm.nih.gov/pubmed/?term=Serruys%20PW%5BAuthor%5D&cauthor=true&cauthor_uid=20965464); [Interventional Cardiologists of the Thoraxcenter](http://www.ncbi.nlm.nih.gov/pubmed/?term=Interventional%20Cardiologists%20of%20the%20Thoraxcenter%5BCorporate%20Author%5D). The unrestricted use of sirolimus- and paclitaxel-eluting stents results in better clinical outcomes during 6-year follow-up than bare-metal stents: an analysis of the RESEARCH (Rapamycin-Eluting Stent Evaluated At Rotterdam Cardiology Hospital) and T-SEARCH (Taxus-Stent Evaluated At Rotterdam Cardiology Hospital) registries. [JACC Cardiovasc Interv.](http://www.ncbi.nlm.nih.gov/pubmed/20965464) 2010 Oct;3(10):1051-8.

1. [Simsek C](http://www.ncbi.nlm.nih.gov/pubmed/?term=Simsek%20C%5BAuthor%5D&cauthor=true&cauthor_uid=20310019)^1^, [Onuma Y](http://www.ncbi.nlm.nih.gov/pubmed/?term=Onuma%20Y%5BAuthor%5D&cauthor=true&cauthor_uid=20310019), [Magro M](http://www.ncbi.nlm.nih.gov/pubmed/?term=Magro%20M%5BAuthor%5D&cauthor=true&cauthor_uid=20310019), [de Boer S](http://www.ncbi.nlm.nih.gov/pubmed/?term=de%20Boer%20S%5BAuthor%5D&cauthor=true&cauthor_uid=20310019), [Battes L](http://www.ncbi.nlm.nih.gov/pubmed/?term=Battes%20L%5BAuthor%5D&cauthor=true&cauthor_uid=20310019), [van Domburg RT](http://www.ncbi.nlm.nih.gov/pubmed/?term=van%20Domburg%20RT%5BAuthor%5D&cauthor=true&cauthor_uid=20310019), [Boersma E](http://www.ncbi.nlm.nih.gov/pubmed/?term=Boersma%20E%5BAuthor%5D&cauthor=true&cauthor_uid=20310019), [Serruys PW](http://www.ncbi.nlm.nih.gov/pubmed/?term=Serruys%20PW%5BAuthor%5D&cauthor=true&cauthor_uid=20310019); [Interventional Cardiologists of the Thoraxcenter (2000-2005)](http://www.ncbi.nlm.nih.gov/pubmed/?term=Interventional%20Cardiologists%20of%20the%20Thoraxcenter%20(2000-2005)%5BCorporate%20Author%5D). Four-year clinical outcome of sirolimus- and paclitaxel-eluting stents compared to bare-metal stents for the percutaneous treatment of stable coronary artery disease. [Catheter Cardiovasc Interv.](http://www.ncbi.nlm.nih.gov/pubmed/20310019) 2010 Jul 1;76(1):41-9.

# [Jensen LO](http://www.ncbi.nlm.nih.gov/pubmed/?term=Jensen%20LO%5BAuthor%5D&cauthor=true&cauthor_uid=20542774)^1^, [Tilsted HH](http://www.ncbi.nlm.nih.gov/pubmed/?term=Tilsted%20HH%5BAuthor%5D&cauthor=true&cauthor_uid=20542774), [Thayssen P](http://www.ncbi.nlm.nih.gov/pubmed/?term=Thayssen%20P%5BAuthor%5D&cauthor=true&cauthor_uid=20542774), [Kaltoft A](http://www.ncbi.nlm.nih.gov/pubmed/?term=Kaltoft%20A%5BAuthor%5D&cauthor=true&cauthor_uid=20542774), [Maeng M](http://www.ncbi.nlm.nih.gov/pubmed/?term=Maeng%20M%5BAuthor%5D&cauthor=true&cauthor_uid=20542774), [Lassen JF](http://www.ncbi.nlm.nih.gov/pubmed/?term=Lassen%20JF%5BAuthor%5D&cauthor=true&cauthor_uid=20542774), [Hansen KN](http://www.ncbi.nlm.nih.gov/pubmed/?term=Hansen%20KN%5BAuthor%5D&cauthor=true&cauthor_uid=20542774), [Madsen M](http://www.ncbi.nlm.nih.gov/pubmed/?term=Madsen%20M%5BAuthor%5D&cauthor=true&cauthor_uid=20542774), [Ravkilde J](http://www.ncbi.nlm.nih.gov/pubmed/?term=Ravkilde%20J%5BAuthor%5D&cauthor=true&cauthor_uid=20542774), [Johnsen SP](http://www.ncbi.nlm.nih.gov/pubmed/?term=Johnsen%20SP%5BAuthor%5D&cauthor=true&cauthor_uid=20542774), [Sørensen HT](http://www.ncbi.nlm.nih.gov/pubmed/?term=S%C3%B8rensen%20HT%5BAuthor%5D&cauthor=true&cauthor_uid=20542774), [Thuesen L](http://www.ncbi.nlm.nih.gov/pubmed/?term=Thuesen%20L%5BAuthor%5D&cauthor=true&cauthor_uid=20542774). Paclitaxel and sirolimus eluting stents versus bare metal stents: long-term risk of stent thrombosis and other outcomes. From the Western Denmark Heart Registry. [EuroIntervention.](http://www.ncbi.nlm.nih.gov/pubmed/20542774) 2010 Apr;5(8):898-905.

# [Gollop ND](http://www.ncbi.nlm.nih.gov/pubmed/?term=Gollop%20ND%5BAuthor%5D&cauthor=true&cauthor_uid=24144807)^1^, [Henderson DB](http://www.ncbi.nlm.nih.gov/pubmed/?term=Henderson%20DB%5BAuthor%5D&cauthor=true&cauthor_uid=24144807), [Flather MD](http://www.ncbi.nlm.nih.gov/pubmed/?term=Flather%20MD%5BAuthor%5D&cauthor=true&cauthor_uid=24144807). Comparison of drug-eluting and bare-metal stents in patients with diabetes undergoing primary percutaneous coronary intervention: what is the evidence? [Interact Cardiovasc Thorac Surg.](http://www.ncbi.nlm.nih.gov/pubmed/24144807) 2014 Jan;18(1):112-6.

# [Brodie BR](http://www.ncbi.nlm.nih.gov/pubmed/?term=Brodie%20BR%5BAuthor%5D&cauthor=true&cauthor_uid=19016465)^1^, [Stuckey T](http://www.ncbi.nlm.nih.gov/pubmed/?term=Stuckey%20T%5BAuthor%5D&cauthor=true&cauthor_uid=19016465), [Downey W](http://www.ncbi.nlm.nih.gov/pubmed/?term=Downey%20W%5BAuthor%5D&cauthor=true&cauthor_uid=19016465), [Humphrey A](http://www.ncbi.nlm.nih.gov/pubmed/?term=Humphrey%20A%5BAuthor%5D&cauthor=true&cauthor_uid=19016465), [Nussbaum M](http://www.ncbi.nlm.nih.gov/pubmed/?term=Nussbaum%20M%5BAuthor%5D&cauthor=true&cauthor_uid=19016465), [Laurent S](http://www.ncbi.nlm.nih.gov/pubmed/?term=Laurent%20S%5BAuthor%5D&cauthor=true&cauthor_uid=19016465), [Bradshaw B](http://www.ncbi.nlm.nih.gov/pubmed/?term=Bradshaw%20B%5BAuthor%5D&cauthor=true&cauthor_uid=19016465), [Metzger C](http://www.ncbi.nlm.nih.gov/pubmed/?term=Metzger%20C%5BAuthor%5D&cauthor=true&cauthor_uid=19016465), [Hermiller J](http://www.ncbi.nlm.nih.gov/pubmed/?term=Hermiller%20J%5BAuthor%5D&cauthor=true&cauthor_uid=19016465), [Krainin F](http://www.ncbi.nlm.nih.gov/pubmed/?term=Krainin%20F%5BAuthor%5D&cauthor=true&cauthor_uid=19016465), [Juk S](http://www.ncbi.nlm.nih.gov/pubmed/?term=Juk%20S%5BAuthor%5D&cauthor=true&cauthor_uid=19016465), [Cheek B](http://www.ncbi.nlm.nih.gov/pubmed/?term=Cheek%20B%5BAuthor%5D&cauthor=true&cauthor_uid=19016465), [Duffy P](http://www.ncbi.nlm.nih.gov/pubmed/?term=Duffy%20P%5BAuthor%5D&cauthor=true&cauthor_uid=19016465), [Simonton CA](http://www.ncbi.nlm.nih.gov/pubmed/?term=Simonton%20CA%5BAuthor%5D&cauthor=true&cauthor_uid=19016465); [Strategic Transcatheter Evaluation of New Therapies (STENT) Group](http://www.ncbi.nlm.nih.gov/pubmed/?term=Strategic%20Transcatheter%20Evaluation%20of%20New%20Therapies%20(STENT)%20Group%5BCorporate%20Author%5D). Outcomes with drug-eluting stents versus bare metal stents in acute ST-elevation myocardial infarction: results from the Strategic Transcatheter Evaluation of New Therapies (STENT) Group. [Catheter Cardiovasc Interv.](http://www.ncbi.nlm.nih.gov/pubmed/19016465) 2008 Dec 1;72(7):893-900.

# [Minha S](http://www.ncbi.nlm.nih.gov/pubmed/?term=Minha%20S%5BAuthor%5D&cauthor=true&cauthor_uid=21805561)^1^, [Bental T](http://www.ncbi.nlm.nih.gov/pubmed/?term=Bental%20T%5BAuthor%5D&cauthor=true&cauthor_uid=21805561), [Assali A](http://www.ncbi.nlm.nih.gov/pubmed/?term=Assali%20A%5BAuthor%5D&cauthor=true&cauthor_uid=21805561), [Vaknin-Assa H](http://www.ncbi.nlm.nih.gov/pubmed/?term=Vaknin-Assa%20H%5BAuthor%5D&cauthor=true&cauthor_uid=21805561), [Lev EI](http://www.ncbi.nlm.nih.gov/pubmed/?term=Lev%20EI%5BAuthor%5D&cauthor=true&cauthor_uid=21805561), [Rechavia E](http://www.ncbi.nlm.nih.gov/pubmed/?term=Rechavia%20E%5BAuthor%5D&cauthor=true&cauthor_uid=21805561), [Battler A](http://www.ncbi.nlm.nih.gov/pubmed/?term=Battler%20A%5BAuthor%5D&cauthor=true&cauthor_uid=21805561), [Kornowski R](http://www.ncbi.nlm.nih.gov/pubmed/?term=Kornowski%20R%5BAuthor%5D&cauthor=true&cauthor_uid=21805561). A comparative analysis of major clinical outcomes using drug-eluting stents versus bare metal stents in diabetic versus nondiabetic patients. [Catheter Cardiovasc Interv.](http://www.ncbi.nlm.nih.gov/pubmed/21805561) 2011 Nov 1;78(5):710-7.

# [Nishio K](http://www.ncbi.nlm.nih.gov/pubmed/?term=Nishio%20K%5BAuthor%5D&cauthor=true&cauthor_uid=21489889)^1^, [Hosaka M](http://www.ncbi.nlm.nih.gov/pubmed/?term=Hosaka%20M%5BAuthor%5D&cauthor=true&cauthor_uid=21489889), [Shigemitsu M](http://www.ncbi.nlm.nih.gov/pubmed/?term=Shigemitsu%20M%5BAuthor%5D&cauthor=true&cauthor_uid=21489889), [Kobayashi Y](http://www.ncbi.nlm.nih.gov/pubmed/?term=Kobayashi%20Y%5BAuthor%5D&cauthor=true&cauthor_uid=21489889). Three-year clinical outcome in type 2 diabetic patients with drug-eluting stents versus bare-metal stents with pioglitazone. [Cardiovasc Revasc Med.](http://www.ncbi.nlm.nih.gov/pubmed/21489889) 2011 Jul-Aug;12(4):197-202.

# [Nishio K](http://www.ncbi.nlm.nih.gov/pubmed/?term=Nishio%20K%5BAuthor%5D&cauthor=true&cauthor_uid=19159848)^1^, [Shigemitsu M](http://www.ncbi.nlm.nih.gov/pubmed/?term=Shigemitsu%20M%5BAuthor%5D&cauthor=true&cauthor_uid=19159848), [Kodama Y](http://www.ncbi.nlm.nih.gov/pubmed/?term=Kodama%20Y%5BAuthor%5D&cauthor=true&cauthor_uid=19159848), [Konno N](http://www.ncbi.nlm.nih.gov/pubmed/?term=Konno%20N%5BAuthor%5D&cauthor=true&cauthor_uid=19159848), [Katagiri T](http://www.ncbi.nlm.nih.gov/pubmed/?term=Katagiri%20T%5BAuthor%5D&cauthor=true&cauthor_uid=19159848), [Kobayashi Y](http://www.ncbi.nlm.nih.gov/pubmed/?term=Kobayashi%20Y%5BAuthor%5D&cauthor=true&cauthor_uid=19159848). Comparison of bare metal stent with pioglitazone versus sirolimus-eluting stent for percutaneous coronary intervention in patients with Type 2 diabetes mellitus. [Cardiovasc Revasc Med.](http://www.ncbi.nlm.nih.gov/pubmed/19159848) 2009 Jan-Mar;10(1):5-11.

# [Koh AS](http://www.ncbi.nlm.nih.gov/pubmed/?term=Koh%20AS%5BAuthor%5D&cauthor=true&cauthor_uid=21164343)^1^, [Chia S](http://www.ncbi.nlm.nih.gov/pubmed/?term=Chia%20S%5BAuthor%5D&cauthor=true&cauthor_uid=21164343), [Choi LM](http://www.ncbi.nlm.nih.gov/pubmed/?term=Choi%20LM%5BAuthor%5D&cauthor=true&cauthor_uid=21164343), [Sim LL](http://www.ncbi.nlm.nih.gov/pubmed/?term=Sim%20LL%5BAuthor%5D&cauthor=true&cauthor_uid=21164343), [Chua TS](http://www.ncbi.nlm.nih.gov/pubmed/?term=Chua%20TS%5BAuthor%5D&cauthor=true&cauthor_uid=21164343), [Koh TH](http://www.ncbi.nlm.nih.gov/pubmed/?term=Koh%20TH%5BAuthor%5D&cauthor=true&cauthor_uid=21164343), [Tan JW](http://www.ncbi.nlm.nih.gov/pubmed/?term=Tan%20JW%5BAuthor%5D&cauthor=true&cauthor_uid=21164343). Long-term outcomes after coronary bare-metal-stent and drug-eluting-stent implantations: a 'real-world' comparison among patients with diabetes with diffuse small vessel coronary artery disease. [Coron Artery Dis.](http://www.ncbi.nlm.nih.gov/pubmed/21164343) 2011 Mar;22(1):96-9.

# [Ramanath VS](http://www.ncbi.nlm.nih.gov/pubmed/?term=Ramanath%20VS%5BAuthor%5D&cauthor=true&cauthor_uid=20882647)^1^, [Brown JR](http://www.ncbi.nlm.nih.gov/pubmed/?term=Brown%20JR%5BAuthor%5D&cauthor=true&cauthor_uid=20882647), [Malenka DJ](http://www.ncbi.nlm.nih.gov/pubmed/?term=Malenka%20DJ%5BAuthor%5D&cauthor=true&cauthor_uid=20882647), [DeVries JT](http://www.ncbi.nlm.nih.gov/pubmed/?term=DeVries%20JT%5BAuthor%5D&cauthor=true&cauthor_uid=20882647), [Sidhu MS](http://www.ncbi.nlm.nih.gov/pubmed/?term=Sidhu%20MS%5BAuthor%5D&cauthor=true&cauthor_uid=20882647), [Robb JF](http://www.ncbi.nlm.nih.gov/pubmed/?term=Robb%20JF%5BAuthor%5D&cauthor=true&cauthor_uid=20882647), [Jayne JE](http://www.ncbi.nlm.nih.gov/pubmed/?term=Jayne%20JE%5BAuthor%5D&cauthor=true&cauthor_uid=20882647), [Hettleman BD](http://www.ncbi.nlm.nih.gov/pubmed/?term=Hettleman%20BD%5BAuthor%5D&cauthor=true&cauthor_uid=20882647), [Friedman BJ](http://www.ncbi.nlm.nih.gov/pubmed/?term=Friedman%20BJ%5BAuthor%5D&cauthor=true&cauthor_uid=20882647), [Niles NW 2nd](http://www.ncbi.nlm.nih.gov/pubmed/?term=Niles%20NW%202nd%5BAuthor%5D&cauthor=true&cauthor_uid=20882647), [Kaplan AV](http://www.ncbi.nlm.nih.gov/pubmed/?term=Kaplan%20AV%5BAuthor%5D&cauthor=true&cauthor_uid=20882647), [Thompson CA](http://www.ncbi.nlm.nih.gov/pubmed/?term=Thompson%20CA%5BAuthor%5D&cauthor=true&cauthor_uid=20882647);[Dartmouth Dynamic Registry Investigators](http://www.ncbi.nlm.nih.gov/pubmed/?term=Dartmouth%20Dynamic%20Registry%20Investigators%5BCorporate%20Author%5D). Outcomes of diabetics receiving bare-metal stents versus drug-eluting stents. [Catheter Cardiovasc Interv.](http://www.ncbi.nlm.nih.gov/pubmed/20882647) 2010 Oct 1;76(4):473-81.

# [De Luca G](http://www.ncbi.nlm.nih.gov/pubmed/?term=De%20Luca%20G%5BAuthor%5D&cauthor=true&cauthor_uid=19921102)^1^, [Sauro R](http://www.ncbi.nlm.nih.gov/pubmed/?term=Sauro%20R%5BAuthor%5D&cauthor=true&cauthor_uid=19921102), [Varricchio A](http://www.ncbi.nlm.nih.gov/pubmed/?term=Varricchio%20A%5BAuthor%5D&cauthor=true&cauthor_uid=19921102), [Capasso M](http://www.ncbi.nlm.nih.gov/pubmed/?term=Capasso%20M%5BAuthor%5D&cauthor=true&cauthor_uid=19921102), [Lanzillo T](http://www.ncbi.nlm.nih.gov/pubmed/?term=Lanzillo%20T%5BAuthor%5D&cauthor=true&cauthor_uid=19921102), [Manganelli F](http://www.ncbi.nlm.nih.gov/pubmed/?term=Manganelli%20F%5BAuthor%5D&cauthor=true&cauthor_uid=19921102), [Mariello C](http://www.ncbi.nlm.nih.gov/pubmed/?term=Mariello%20C%5BAuthor%5D&cauthor=true&cauthor_uid=19921102), [Siano F](http://www.ncbi.nlm.nih.gov/pubmed/?term=Siano%20F%5BAuthor%5D&cauthor=true&cauthor_uid=19921102), [Carbone G](http://www.ncbi.nlm.nih.gov/pubmed/?term=Carbone%20G%5BAuthor%5D&cauthor=true&cauthor_uid=19921102), [Pagliuca MR](http://www.ncbi.nlm.nih.gov/pubmed/?term=Pagliuca%20MR%5BAuthor%5D&cauthor=true&cauthor_uid=19921102), [Rosato G](http://www.ncbi.nlm.nih.gov/pubmed/?term=Rosato%20G%5BAuthor%5D&cauthor=true&cauthor_uid=19921102), [Di Lorenzo E](http://www.ncbi.nlm.nih.gov/pubmed/?term=Di%20Lorenzo%20E%5BAuthor%5D&cauthor=true&cauthor_uid=19921102). Impact of diabetes on long-term outcome in STEMI patients undergoing primary angioplasty with glycoprotein IIb-IIIa inhibitors and BMS or DES. [J Thromb Thrombolysis.](http://www.ncbi.nlm.nih.gov/pubmed/19921102) 2010 Aug;30(2):133-41.

# [Stenestrand U](http://www.ncbi.nlm.nih.gov/pubmed/?term=Stenestrand%20U%5BAuthor%5D&cauthor=true&cauthor_uid=19903684)^1^, [James SK](http://www.ncbi.nlm.nih.gov/pubmed/?term=James%20SK%5BAuthor%5D&cauthor=true&cauthor_uid=19903684), [Lindbäck J](http://www.ncbi.nlm.nih.gov/pubmed/?term=Lindb%C3%A4ck%20J%5BAuthor%5D&cauthor=true&cauthor_uid=19903684), [Fröbert O](http://www.ncbi.nlm.nih.gov/pubmed/?term=Fr%C3%B6bert%20O%5BAuthor%5D&cauthor=true&cauthor_uid=19903684), [Carlsson J](http://www.ncbi.nlm.nih.gov/pubmed/?term=Carlsson%20J%5BAuthor%5D&cauthor=true&cauthor_uid=19903684), [Scherstén F](http://www.ncbi.nlm.nih.gov/pubmed/?term=Scherst%C3%A9n%20F%5BAuthor%5D&cauthor=true&cauthor_uid=19903684), [Nilsson T](http://www.ncbi.nlm.nih.gov/pubmed/?term=Nilsson%20T%5BAuthor%5D&cauthor=true&cauthor_uid=19903684), [Lagerqvist B](http://www.ncbi.nlm.nih.gov/pubmed/?term=Lagerqvist%20B%5BAuthor%5D&cauthor=true&cauthor_uid=19903684); [SCAAR/SWEDEHEART study group](http://www.ncbi.nlm.nih.gov/pubmed/?term=SCAAR%2FSWEDEHEART%20study%20group%5BCorporate%20Author%5D). Safety and efficacy of drug-eluting vs. bare metal stents in patients with diabetes mellitus: long-term follow-up in the Swedish Coronary Angiography and Angioplasty Registry (SCAAR). [Eur Heart J.](http://www.ncbi.nlm.nih.gov/pubmed/19903684) 2010 Jan;31(2):177-86.

# [Dou KF](http://www.ncbi.nlm.nih.gov/pubmed/?term=Dou%20KF%5BAuthor%5D&cauthor=true&cauthor_uid=19323921)^1^, [Xu B](http://www.ncbi.nlm.nih.gov/pubmed/?term=Xu%20B%5BAuthor%5D&cauthor=true&cauthor_uid=19323921), [Yang YJ](http://www.ncbi.nlm.nih.gov/pubmed/?term=Yang%20YJ%5BAuthor%5D&cauthor=true&cauthor_uid=19323921), [Chen JL](http://www.ncbi.nlm.nih.gov/pubmed/?term=Chen%20JL%5BAuthor%5D&cauthor=true&cauthor_uid=19323921), [Qiao SB](http://www.ncbi.nlm.nih.gov/pubmed/?term=Qiao%20SB%5BAuthor%5D&cauthor=true&cauthor_uid=19323921), [Li JJ](http://www.ncbi.nlm.nih.gov/pubmed/?term=Li%20JJ%5BAuthor%5D&cauthor=true&cauthor_uid=19323921), [Qin XW](http://www.ncbi.nlm.nih.gov/pubmed/?term=Qin%20XW%5BAuthor%5D&cauthor=true&cauthor_uid=19323921), [Liu HB](http://www.ncbi.nlm.nih.gov/pubmed/?term=Liu%20HB%5BAuthor%5D&cauthor=true&cauthor_uid=19323921), [Wu YJ](http://www.ncbi.nlm.nih.gov/pubmed/?term=Wu%20YJ%5BAuthor%5D&cauthor=true&cauthor_uid=19323921), [Chen J](http://www.ncbi.nlm.nih.gov/pubmed/?term=Chen%20J%5BAuthor%5D&cauthor=true&cauthor_uid=19323921), [Yao M](http://www.ncbi.nlm.nih.gov/pubmed/?term=Yao%20M%5BAuthor%5D&cauthor=true&cauthor_uid=19323921), [You SJ](http://www.ncbi.nlm.nih.gov/pubmed/?term=You%20SJ%5BAuthor%5D&cauthor=true&cauthor_uid=19323921), [Yuan JQ](http://www.ncbi.nlm.nih.gov/pubmed/?term=Yuan%20JQ%5BAuthor%5D&cauthor=true&cauthor_uid=19323921), [Dai J](http://www.ncbi.nlm.nih.gov/pubmed/?term=Dai%20J%5BAuthor%5D&cauthor=true&cauthor_uid=19323921), [Gao RL](http://www.ncbi.nlm.nih.gov/pubmed/?term=Gao%20RL%5BAuthor%5D&cauthor=true&cauthor_uid=19323921). Two-year clinical outcome after successful implantation of drug-eluting and bare metal stents in diabetic patients: results from a real-world single center registry. [Chin Med J (Engl).](http://www.ncbi.nlm.nih.gov/pubmed/19323921) 2009 Mar 20;122(6):612-6.

# [Capodanno D](http://www.ncbi.nlm.nih.gov/pubmed/?term=Capodanno%20D%5BAuthor%5D&cauthor=true&cauthor_uid=19214964)^1^, [Di Salvo ME](http://www.ncbi.nlm.nih.gov/pubmed/?term=Di%20Salvo%20ME%5BAuthor%5D&cauthor=true&cauthor_uid=19214964), [Palmerini T](http://www.ncbi.nlm.nih.gov/pubmed/?term=Palmerini%20T%5BAuthor%5D&cauthor=true&cauthor_uid=19214964), [Sheiban I](http://www.ncbi.nlm.nih.gov/pubmed/?term=Sheiban%20I%5BAuthor%5D&cauthor=true&cauthor_uid=19214964), [Margheri M](http://www.ncbi.nlm.nih.gov/pubmed/?term=Margheri%20M%5BAuthor%5D&cauthor=true&cauthor_uid=19214964), [Vecchi G](http://www.ncbi.nlm.nih.gov/pubmed/?term=Vecchi%20G%5BAuthor%5D&cauthor=true&cauthor_uid=19214964), [Sangiorgi G](http://www.ncbi.nlm.nih.gov/pubmed/?term=Sangiorgi%20G%5BAuthor%5D&cauthor=true&cauthor_uid=19214964), [Piovaccari G](http://www.ncbi.nlm.nih.gov/pubmed/?term=Piovaccari%20G%5BAuthor%5D&cauthor=true&cauthor_uid=19214964), [Bartorelli A](http://www.ncbi.nlm.nih.gov/pubmed/?term=Bartorelli%20A%5BAuthor%5D&cauthor=true&cauthor_uid=19214964), [Briguori C](http://www.ncbi.nlm.nih.gov/pubmed/?term=Briguori%20C%5BAuthor%5D&cauthor=true&cauthor_uid=19214964), [Ardissino D](http://www.ncbi.nlm.nih.gov/pubmed/?term=Ardissino%20D%5BAuthor%5D&cauthor=true&cauthor_uid=19214964), [Di Pede F](http://www.ncbi.nlm.nih.gov/pubmed/?term=Di%20Pede%20F%5BAuthor%5D&cauthor=true&cauthor_uid=19214964),[Ramondo A](http://www.ncbi.nlm.nih.gov/pubmed/?term=Ramondo%20A%5BAuthor%5D&cauthor=true&cauthor_uid=19214964), [Inglese L](http://www.ncbi.nlm.nih.gov/pubmed/?term=Inglese%20L%5BAuthor%5D&cauthor=true&cauthor_uid=19214964), [Petronio AS](http://www.ncbi.nlm.nih.gov/pubmed/?term=Petronio%20AS%5BAuthor%5D&cauthor=true&cauthor_uid=19214964), [Bolognese L](http://www.ncbi.nlm.nih.gov/pubmed/?term=Bolognese%20L%5BAuthor%5D&cauthor=true&cauthor_uid=19214964), [Benassi A](http://www.ncbi.nlm.nih.gov/pubmed/?term=Benassi%20A%5BAuthor%5D&cauthor=true&cauthor_uid=19214964), [Palmieri C](http://www.ncbi.nlm.nih.gov/pubmed/?term=Palmieri%20C%5BAuthor%5D&cauthor=true&cauthor_uid=19214964), [Filippone V](http://www.ncbi.nlm.nih.gov/pubmed/?term=Filippone%20V%5BAuthor%5D&cauthor=true&cauthor_uid=19214964), [De Servi S](http://www.ncbi.nlm.nih.gov/pubmed/?term=De%20Servi%20S%5BAuthor%5D&cauthor=true&cauthor_uid=19214964), [Tamburino C](http://www.ncbi.nlm.nih.gov/pubmed/?term=Tamburino%20C%5BAuthor%5D&cauthor=true&cauthor_uid=19214964). Long-term clinical benefit of drug-eluting stents over bare-metal stents in diabetic patients with de novo left main coronary artery disease: results from a real-world multicenter registry. [Catheter Cardiovasc Interv.](http://www.ncbi.nlm.nih.gov/pubmed/19214964) 2009 Feb 15;73(3):310-6.

# [Garg P](http://www.ncbi.nlm.nih.gov/pubmed/?term=Garg%20P%5BAuthor%5D&cauthor=true&cauthor_uid=19001019)^1^, [Normand SL](http://www.ncbi.nlm.nih.gov/pubmed/?term=Normand%20SL%5BAuthor%5D&cauthor=true&cauthor_uid=19001019), [Silbaugh TS](http://www.ncbi.nlm.nih.gov/pubmed/?term=Silbaugh%20TS%5BAuthor%5D&cauthor=true&cauthor_uid=19001019), [Wolf RE](http://www.ncbi.nlm.nih.gov/pubmed/?term=Wolf%20RE%5BAuthor%5D&cauthor=true&cauthor_uid=19001019), [Zelevinsky K](http://www.ncbi.nlm.nih.gov/pubmed/?term=Zelevinsky%20K%5BAuthor%5D&cauthor=true&cauthor_uid=19001019), [Lovett A](http://www.ncbi.nlm.nih.gov/pubmed/?term=Lovett%20A%5BAuthor%5D&cauthor=true&cauthor_uid=19001019), [Varma MR](http://www.ncbi.nlm.nih.gov/pubmed/?term=Varma%20MR%5BAuthor%5D&cauthor=true&cauthor_uid=19001019), [Zhou Z](http://www.ncbi.nlm.nih.gov/pubmed/?term=Zhou%20Z%5BAuthor%5D&cauthor=true&cauthor_uid=19001019), [Mauri L](http://www.ncbi.nlm.nih.gov/pubmed/?term=Mauri%20L%5BAuthor%5D&cauthor=true&cauthor_uid=19001019). Drug-eluting or bare-metal stenting in patients with diabetes mellitus: results from the Massachusetts Data Analysis Center Registry. [Circulation.](http://www.ncbi.nlm.nih.gov/pubmed/19001019) 2008 Nov 25;118(22):2277-85, 7p following 2285.

# [Maeng M](http://www.ncbi.nlm.nih.gov/pubmed/?term=Maeng%20M%5BAuthor%5D&cauthor=true&cauthor_uid=18602515)^1^, [Jensen LO](http://www.ncbi.nlm.nih.gov/pubmed/?term=Jensen%20LO%5BAuthor%5D&cauthor=true&cauthor_uid=18602515), [Kaltoft A](http://www.ncbi.nlm.nih.gov/pubmed/?term=Kaltoft%20A%5BAuthor%5D&cauthor=true&cauthor_uid=18602515), [Hansen HH](http://www.ncbi.nlm.nih.gov/pubmed/?term=Hansen%20HH%5BAuthor%5D&cauthor=true&cauthor_uid=18602515), [Bøttcher M](http://www.ncbi.nlm.nih.gov/pubmed/?term=B%C3%B8ttcher%20M%5BAuthor%5D&cauthor=true&cauthor_uid=18602515), [Lassen JF](http://www.ncbi.nlm.nih.gov/pubmed/?term=Lassen%20JF%5BAuthor%5D&cauthor=true&cauthor_uid=18602515), [Thayssen P](http://www.ncbi.nlm.nih.gov/pubmed/?term=Thayssen%20P%5BAuthor%5D&cauthor=true&cauthor_uid=18602515), [Krusell LR](http://www.ncbi.nlm.nih.gov/pubmed/?term=Krusell%20LR%5BAuthor%5D&cauthor=true&cauthor_uid=18602515), [Rasmussen K](http://www.ncbi.nlm.nih.gov/pubmed/?term=Rasmussen%20K%5BAuthor%5D&cauthor=true&cauthor_uid=18602515), [Pedersen L](http://www.ncbi.nlm.nih.gov/pubmed/?term=Pedersen%20L%5BAuthor%5D&cauthor=true&cauthor_uid=18602515), [Sørensen HT](http://www.ncbi.nlm.nih.gov/pubmed/?term=S%C3%B8rensen%20HT%5BAuthor%5D&cauthor=true&cauthor_uid=18602515), [Johnsen SP](http://www.ncbi.nlm.nih.gov/pubmed/?term=Johnsen%20SP%5BAuthor%5D&cauthor=true&cauthor_uid=18602515), [Thuesen L](http://www.ncbi.nlm.nih.gov/pubmed/?term=Thuesen%20L%5BAuthor%5D&cauthor=true&cauthor_uid=18602515). Comparison of stent thrombosis, myocardial infarction, and mortality following drug-eluting versus bare-metal stent coronary intervention in patients with diabetes mellitus. [Am J Cardiol.](http://www.ncbi.nlm.nih.gov/pubmed/18602515) 2008 Jul 15;102(2):165-72.

# [Qiao SB](http://www.ncbi.nlm.nih.gov/pubmed/?term=Qiao%20SB%5BAuthor%5D&cauthor=true&cauthor_uid=17711711)^1^, [Hou Q](http://www.ncbi.nlm.nih.gov/pubmed/?term=Hou%20Q%5BAuthor%5D&cauthor=true&cauthor_uid=17711711), [Xu B](http://www.ncbi.nlm.nih.gov/pubmed/?term=Xu%20B%5BAuthor%5D&cauthor=true&cauthor_uid=17711711), [Chen J](http://www.ncbi.nlm.nih.gov/pubmed/?term=Chen%20J%5BAuthor%5D&cauthor=true&cauthor_uid=17711711), [Liu HB](http://www.ncbi.nlm.nih.gov/pubmed/?term=Liu%20HB%5BAuthor%5D&cauthor=true&cauthor_uid=17711711), [Yang YJ](http://www.ncbi.nlm.nih.gov/pubmed/?term=Yang%20YJ%5BAuthor%5D&cauthor=true&cauthor_uid=17711711), [Wu YJ](http://www.ncbi.nlm.nih.gov/pubmed/?term=Wu%20YJ%5BAuthor%5D&cauthor=true&cauthor_uid=17711711), [Yuan JQ](http://www.ncbi.nlm.nih.gov/pubmed/?term=Yuan%20JQ%5BAuthor%5D&cauthor=true&cauthor_uid=17711711), [Wu Y](http://www.ncbi.nlm.nih.gov/pubmed/?term=Wu%20Y%5BAuthor%5D&cauthor=true&cauthor_uid=17711711), [Dai J](http://www.ncbi.nlm.nih.gov/pubmed/?term=Dai%20J%5BAuthor%5D&cauthor=true&cauthor_uid=17711711), [You SJ](http://www.ncbi.nlm.nih.gov/pubmed/?term=You%20SJ%5BAuthor%5D&cauthor=true&cauthor_uid=17711711), [Ma WH](http://www.ncbi.nlm.nih.gov/pubmed/?term=Ma%20WH%5BAuthor%5D&cauthor=true&cauthor_uid=17711711), [Zhang P](http://www.ncbi.nlm.nih.gov/pubmed/?term=Zhang%20P%5BAuthor%5D&cauthor=true&cauthor_uid=17711711), [Gao Z](http://www.ncbi.nlm.nih.gov/pubmed/?term=Gao%20Z%5BAuthor%5D&cauthor=true&cauthor_uid=17711711), [Dou KF](http://www.ncbi.nlm.nih.gov/pubmed/?term=Dou%20KF%5BAuthor%5D&cauthor=true&cauthor_uid=17711711), [Qiu H](http://www.ncbi.nlm.nih.gov/pubmed/?term=Qiu%20H%5BAuthor%5D&cauthor=true&cauthor_uid=17711711), [Mu CW](http://www.ncbi.nlm.nih.gov/pubmed/?term=Mu%20CW%5BAuthor%5D&cauthor=true&cauthor_uid=17711711), [Chen JL](http://www.ncbi.nlm.nih.gov/pubmed/?term=Chen%20JL%5BAuthor%5D&cauthor=true&cauthor_uid=17711711), [Gao RL](http://www.ncbi.nlm.nih.gov/pubmed/?term=Gao%20RL%5BAuthor%5D&cauthor=true&cauthor_uid=17711711). [Clinical and angiographic outcome in coronary artery disease patients with type-II diabetes mellitus undergoing elective bare-metal stenting or drug-eluting stenting]. [Zhonghua Xin Xue Guan Bing Za Zhi.](http://www.ncbi.nlm.nih.gov/pubmed/17711711) 2007 Jun;35(6):523-6.

# [Daemen J](http://www.ncbi.nlm.nih.gov/pubmed/?term=Daemen%20J%5BAuthor%5D&cauthor=true&cauthor_uid=17135282)^1^, [Garcia-Garcia HM](http://www.ncbi.nlm.nih.gov/pubmed/?term=Garcia-Garcia%20HM%5BAuthor%5D&cauthor=true&cauthor_uid=17135282), [Kukreja N](http://www.ncbi.nlm.nih.gov/pubmed/?term=Kukreja%20N%5BAuthor%5D&cauthor=true&cauthor_uid=17135282), [Imani F](http://www.ncbi.nlm.nih.gov/pubmed/?term=Imani%20F%5BAuthor%5D&cauthor=true&cauthor_uid=17135282), [de Jaegere PP](http://www.ncbi.nlm.nih.gov/pubmed/?term=de%20Jaegere%20PP%5BAuthor%5D&cauthor=true&cauthor_uid=17135282), [Sianos G](http://www.ncbi.nlm.nih.gov/pubmed/?term=Sianos%20G%5BAuthor%5D&cauthor=true&cauthor_uid=17135282), [van Domburg RT](http://www.ncbi.nlm.nih.gov/pubmed/?term=van%20Domburg%20RT%5BAuthor%5D&cauthor=true&cauthor_uid=17135282), [Serruys PW](http://www.ncbi.nlm.nih.gov/pubmed/?term=Serruys%20PW%5BAuthor%5D&cauthor=true&cauthor_uid=17135282). The long-term value of sirolimus- and paclitaxel-eluting stents over bare metal stents in patients with diabetes mellitus. [Eur Heart J.](http://www.ncbi.nlm.nih.gov/pubmed/17135282) 2007 Jan;28(1):26-32. Epub 2006 Nov 29.

# [Nishio K](http://www.ncbi.nlm.nih.gov/pubmed/?term=Nishio%20K%5BAuthor%5D&cauthor=true&cauthor_uid=19159848)^1^, [Shigemitsu M](http://www.ncbi.nlm.nih.gov/pubmed/?term=Shigemitsu%20M%5BAuthor%5D&cauthor=true&cauthor_uid=19159848), [Kodama Y](http://www.ncbi.nlm.nih.gov/pubmed/?term=Kodama%20Y%5BAuthor%5D&cauthor=true&cauthor_uid=19159848), [Konno N](http://www.ncbi.nlm.nih.gov/pubmed/?term=Konno%20N%5BAuthor%5D&cauthor=true&cauthor_uid=19159848), [Katagiri T](http://www.ncbi.nlm.nih.gov/pubmed/?term=Katagiri%20T%5BAuthor%5D&cauthor=true&cauthor_uid=19159848), [Kobayashi Y](http://www.ncbi.nlm.nih.gov/pubmed/?term=Kobayashi%20Y%5BAuthor%5D&cauthor=true&cauthor_uid=19159848). Comparison of bare metal stent with pioglitazone versus sirolimus-eluting stent for percutaneous coronaryintervention in patients with Type 2 diabetes mellitus. [Cardiovasc Revasc Med.](http://www.ncbi.nlm.nih.gov/pubmed/?term=Comparison+of+bare+metal+stent+with+pioglitazone+versus+sirolimus-eluting+stent+for+percutaneous+coronary+intervention+in+patients+with+Type+2+diabetes+mellitus) 2009 Jan-Mar;10(1):5-11.

# [Yoshida T](http://www.ncbi.nlm.nih.gov/pubmed/?term=Yoshida%20T%5BAuthor%5D&cauthor=true&cauthor_uid=25758470)^1^, [Sakata K](http://www.ncbi.nlm.nih.gov/pubmed/?term=Sakata%20K%5BAuthor%5D&cauthor=true&cauthor_uid=25758470), [Nitta Y](http://www.ncbi.nlm.nih.gov/pubmed/?term=Nitta%20Y%5BAuthor%5D&cauthor=true&cauthor_uid=25758470), [Taguchi T](http://www.ncbi.nlm.nih.gov/pubmed/?term=Taguchi%20T%5BAuthor%5D&cauthor=true&cauthor_uid=25758470), [Kaku B](http://www.ncbi.nlm.nih.gov/pubmed/?term=Kaku%20B%5BAuthor%5D&cauthor=true&cauthor_uid=25758470), [Katsuda S](http://www.ncbi.nlm.nih.gov/pubmed/?term=Katsuda%20S%5BAuthor%5D&cauthor=true&cauthor_uid=25758470), [Shimojima M](http://www.ncbi.nlm.nih.gov/pubmed/?term=Shimojima%20M%5BAuthor%5D&cauthor=true&cauthor_uid=25758470), [Gamou T](http://www.ncbi.nlm.nih.gov/pubmed/?term=Gamou%20T%5BAuthor%5D&cauthor=true&cauthor_uid=25758470), [Nakahashi T](http://www.ncbi.nlm.nih.gov/pubmed/?term=Nakahashi%20T%5BAuthor%5D&cauthor=true&cauthor_uid=25758470), [Konno T](http://www.ncbi.nlm.nih.gov/pubmed/?term=Konno%20T%5BAuthor%5D&cauthor=true&cauthor_uid=25758470), [Kawashiri MA](http://www.ncbi.nlm.nih.gov/pubmed/?term=Kawashiri%20MA%5BAuthor%5D&cauthor=true&cauthor_uid=25758470), [Yamagishi M](http://www.ncbi.nlm.nih.gov/pubmed/?term=Yamagishi%20M%5BAuthor%5D&cauthor=true&cauthor_uid=25758470), [Hayashi K](http://www.ncbi.nlm.nih.gov/pubmed/?term=Hayashi%20K%5BAuthor%5D&cauthor=true&cauthor_uid=25758470). Short- and long-term benefits of drug-eluting stents compared to bare metal stents even in treatment for large coronary arteries. [Heart Vessels.](http://www.ncbi.nlm.nih.gov/pubmed/25758470) 2015 Mar 11.

# [Jing J](http://www.ncbi.nlm.nih.gov/pubmed/?term=Jing%20J%5BAuthor%5D&cauthor=true&cauthor_uid=25629542)^1^. Efficacy and safety of sirolimus-eluting stents versus bare-metal stents in coronary artery disease patients with diabetes. [Cardiovasc J Afr.](http://www.ncbi.nlm.nih.gov/pubmed/25629542) 2014 Sep-Oct;25(5):249.

1. [Assali A](http://www.ncbi.nlm.nih.gov/pubmed/?term=Assali%20A%5BAuthor%5D&cauthor=true&cauthor_uid=22440117)^1^, [Vaduganathan M](http://www.ncbi.nlm.nih.gov/pubmed/?term=Vaduganathan%20M%5BAuthor%5D&cauthor=true&cauthor_uid=22440117), [Vaknin-Assa H](http://www.ncbi.nlm.nih.gov/pubmed/?term=Vaknin-Assa%20H%5BAuthor%5D&cauthor=true&cauthor_uid=22440117), [Lev EI](http://www.ncbi.nlm.nih.gov/pubmed/?term=Lev%20EI%5BAuthor%5D&cauthor=true&cauthor_uid=22440117), [Brosh D](http://www.ncbi.nlm.nih.gov/pubmed/?term=Brosh%20D%5BAuthor%5D&cauthor=true&cauthor_uid=22440117), [Teplitsky I](http://www.ncbi.nlm.nih.gov/pubmed/?term=Teplitsky%20I%5BAuthor%5D&cauthor=true&cauthor_uid=22440117), [Bental T](http://www.ncbi.nlm.nih.gov/pubmed/?term=Bental%20T%5BAuthor%5D&cauthor=true&cauthor_uid=22440117), [Battler A](http://www.ncbi.nlm.nih.gov/pubmed/?term=Battler%20A%5BAuthor%5D&cauthor=true&cauthor_uid=22440117), [Kornowski R](http://www.ncbi.nlm.nih.gov/pubmed/?term=Kornowski%20R%5BAuthor%5D&cauthor=true&cauthor_uid=22440117). Comparison of late (3-year) registry data outcomes using bare metal versus drug-eluting stents for treating ST-segment elevation acute myocardial infarctions. [Am J Cardiol.](http://www.ncbi.nlm.nih.gov/pubmed/22440117) 2012 Jun 1;109(11):1563-8.

# [Manari A](http://www.ncbi.nlm.nih.gov/pubmed/?term=Manari%20A%5BAuthor%5D&cauthor=true&cauthor_uid=20814313)^1^, [Ortolani P](http://www.ncbi.nlm.nih.gov/pubmed/?term=Ortolani%20P%5BAuthor%5D&cauthor=true&cauthor_uid=20814313), [Guastaroba P](http://www.ncbi.nlm.nih.gov/pubmed/?term=Guastaroba%20P%5BAuthor%5D&cauthor=true&cauthor_uid=20814313), [Marzaroli P](http://www.ncbi.nlm.nih.gov/pubmed/?term=Marzaroli%20P%5BAuthor%5D&cauthor=true&cauthor_uid=20814313), [Menozzi M](http://www.ncbi.nlm.nih.gov/pubmed/?term=Menozzi%20M%5BAuthor%5D&cauthor=true&cauthor_uid=20814313), [Magnavacchi P](http://www.ncbi.nlm.nih.gov/pubmed/?term=Magnavacchi%20P%5BAuthor%5D&cauthor=true&cauthor_uid=20814313), [Varani E](http://www.ncbi.nlm.nih.gov/pubmed/?term=Varani%20E%5BAuthor%5D&cauthor=true&cauthor_uid=20814313), [Vignali L](http://www.ncbi.nlm.nih.gov/pubmed/?term=Vignali%20L%5BAuthor%5D&cauthor=true&cauthor_uid=20814313), [Campo G](http://www.ncbi.nlm.nih.gov/pubmed/?term=Campo%20G%5BAuthor%5D&cauthor=true&cauthor_uid=20814313), [Marzocchi A](http://www.ncbi.nlm.nih.gov/pubmed/?term=Marzocchi%20A%5BAuthor%5D&cauthor=true&cauthor_uid=20814313). Long-term outcomes with cobalt-chromium bare-metal vs. drug-eluting stents: the REgistro regionale AngiopLastiche dell'Emilia-Romagna registry. [J Cardiovasc Med (Hagerstown).](http://www.ncbi.nlm.nih.gov/pubmed/20814313) 2011 Feb;12(2):102-9.

# [Maresta A](http://www.ncbi.nlm.nih.gov/pubmed/?term=Maresta%20A%5BAuthor%5D&cauthor=true&cauthor_uid=18489933)^1^, [Varani E](http://www.ncbi.nlm.nih.gov/pubmed/?term=Varani%20E%5BAuthor%5D&cauthor=true&cauthor_uid=18489933), [Balducelli M](http://www.ncbi.nlm.nih.gov/pubmed/?term=Balducelli%20M%5BAuthor%5D&cauthor=true&cauthor_uid=18489933), [Varbella F](http://www.ncbi.nlm.nih.gov/pubmed/?term=Varbella%20F%5BAuthor%5D&cauthor=true&cauthor_uid=18489933), [Lettieri C](http://www.ncbi.nlm.nih.gov/pubmed/?term=Lettieri%20C%5BAuthor%5D&cauthor=true&cauthor_uid=18489933), [Uguccioni L](http://www.ncbi.nlm.nih.gov/pubmed/?term=Uguccioni%20L%5BAuthor%5D&cauthor=true&cauthor_uid=18489933), [Sangiorgio P](http://www.ncbi.nlm.nih.gov/pubmed/?term=Sangiorgio%20P%5BAuthor%5D&cauthor=true&cauthor_uid=18489933), [Zoccai GB](http://www.ncbi.nlm.nih.gov/pubmed/?term=Zoccai%20GB%5BAuthor%5D&cauthor=true&cauthor_uid=18489933); [DESSERT Investigators](http://www.ncbi.nlm.nih.gov/pubmed/?term=DESSERT%20Investigators%5BCorporate%20Author%5D). Comparison of effectiveness and safety of sirolimus-eluting stents versus bare-metal stents in patients withdiabetes mellitus (from the Italian Multicenter Randomized DESSERT Study). [Am J Cardiol.](http://www.ncbi.nlm.nih.gov/pubmed/18489933) 2008 Jun 1;101(11):1560-6.

# [Aoki J](http://www.ncbi.nlm.nih.gov/pubmed/?term=Aoki%20J%5BAuthor%5D&cauthor=true&cauthor_uid=16003016)^1^, [Ong A](http://www.ncbi.nlm.nih.gov/pubmed/?term=Ong%20A%5BAuthor%5D&cauthor=true&cauthor_uid=16003016), [Rodriguez-Granillo G](http://www.ncbi.nlm.nih.gov/pubmed/?term=Rodriguez-Granillo%20G%5BAuthor%5D&cauthor=true&cauthor_uid=16003016), [VanMieghem C](http://www.ncbi.nlm.nih.gov/pubmed/?term=VanMieghem%20C%5BAuthor%5D&cauthor=true&cauthor_uid=16003016), [Daemen J](http://www.ncbi.nlm.nih.gov/pubmed/?term=Daemen%20J%5BAuthor%5D&cauthor=true&cauthor_uid=16003016), [Sonnenschein K](http://www.ncbi.nlm.nih.gov/pubmed/?term=Sonnenschein%20K%5BAuthor%5D&cauthor=true&cauthor_uid=16003016), [McFadden E](http://www.ncbi.nlm.nih.gov/pubmed/?term=McFadden%20E%5BAuthor%5D&cauthor=true&cauthor_uid=16003016), [Sianos G](http://www.ncbi.nlm.nih.gov/pubmed/?term=Sianos%20G%5BAuthor%5D&cauthor=true&cauthor_uid=16003016), [van der Giessen W](http://www.ncbi.nlm.nih.gov/pubmed/?term=van%20der%20Giessen%20W%5BAuthor%5D&cauthor=true&cauthor_uid=16003016), [de Feyter P](http://www.ncbi.nlm.nih.gov/pubmed/?term=de%20Feyter%20P%5BAuthor%5D&cauthor=true&cauthor_uid=16003016), [van Domburg R](http://www.ncbi.nlm.nih.gov/pubmed/?term=van%20Domburg%20R%5BAuthor%5D&cauthor=true&cauthor_uid=16003016),[Serruys P](http://www.ncbi.nlm.nih.gov/pubmed/?term=Serruys%20P%5BAuthor%5D&cauthor=true&cauthor_uid=16003016). The efficacy of sirolimus-eluting stents versus bare metal stents for diabetic patients undergoing elective percutaneous coronary intervention. [J Invasive Cardiol.](http://www.ncbi.nlm.nih.gov/pubmed/16003016) 2005 Jul;17(7):344-8.

# ***10 more articles were excluded because they were meta-analyses:***

# [Stettler C](http://www.ncbi.nlm.nih.gov/pubmed/?term=Stettler%20C%5BAuthor%5D&cauthor=true&cauthor_uid=18757996)^1^, [Allemann S](http://www.ncbi.nlm.nih.gov/pubmed/?term=Allemann%20S%5BAuthor%5D&cauthor=true&cauthor_uid=18757996), [Wandel S](http://www.ncbi.nlm.nih.gov/pubmed/?term=Wandel%20S%5BAuthor%5D&cauthor=true&cauthor_uid=18757996), [Kastrati A](http://www.ncbi.nlm.nih.gov/pubmed/?term=Kastrati%20A%5BAuthor%5D&cauthor=true&cauthor_uid=18757996), [Morice MC](http://www.ncbi.nlm.nih.gov/pubmed/?term=Morice%20MC%5BAuthor%5D&cauthor=true&cauthor_uid=18757996), [Schömig A](http://www.ncbi.nlm.nih.gov/pubmed/?term=Sch%C3%B6mig%20A%5BAuthor%5D&cauthor=true&cauthor_uid=18757996), [Pfisterer ME](http://www.ncbi.nlm.nih.gov/pubmed/?term=Pfisterer%20ME%5BAuthor%5D&cauthor=true&cauthor_uid=18757996), [Stone GW](http://www.ncbi.nlm.nih.gov/pubmed/?term=Stone%20GW%5BAuthor%5D&cauthor=true&cauthor_uid=18757996), [Leon MB](http://www.ncbi.nlm.nih.gov/pubmed/?term=Leon%20MB%5BAuthor%5D&cauthor=true&cauthor_uid=18757996), [de Lezo JS](http://www.ncbi.nlm.nih.gov/pubmed/?term=de%20Lezo%20JS%5BAuthor%5D&cauthor=true&cauthor_uid=18757996), [Goy JJ](http://www.ncbi.nlm.nih.gov/pubmed/?term=Goy%20JJ%5BAuthor%5D&cauthor=true&cauthor_uid=18757996), [Park SJ](http://www.ncbi.nlm.nih.gov/pubmed/?term=Park%20SJ%5BAuthor%5D&cauthor=true&cauthor_uid=18757996), [Sabaté M](http://www.ncbi.nlm.nih.gov/pubmed/?term=Sabat%C3%A9%20M%5BAuthor%5D&cauthor=true&cauthor_uid=18757996), [Suttorp MJ](http://www.ncbi.nlm.nih.gov/pubmed/?term=Suttorp%20MJ%5BAuthor%5D&cauthor=true&cauthor_uid=18757996),[Kelbaek H](http://www.ncbi.nlm.nih.gov/pubmed/?term=Kelbaek%20H%5BAuthor%5D&cauthor=true&cauthor_uid=18757996), [Spaulding C](http://www.ncbi.nlm.nih.gov/pubmed/?term=Spaulding%20C%5BAuthor%5D&cauthor=true&cauthor_uid=18757996), [Menichelli M](http://www.ncbi.nlm.nih.gov/pubmed/?term=Menichelli%20M%5BAuthor%5D&cauthor=true&cauthor_uid=18757996), [Vermeersch P](http://www.ncbi.nlm.nih.gov/pubmed/?term=Vermeersch%20P%5BAuthor%5D&cauthor=true&cauthor_uid=18757996), [Dirksen MT](http://www.ncbi.nlm.nih.gov/pubmed/?term=Dirksen%20MT%5BAuthor%5D&cauthor=true&cauthor_uid=18757996), [Cervinka P](http://www.ncbi.nlm.nih.gov/pubmed/?term=Cervinka%20P%5BAuthor%5D&cauthor=true&cauthor_uid=18757996), [De Carlo M](http://www.ncbi.nlm.nih.gov/pubmed/?term=De%20Carlo%20M%5BAuthor%5D&cauthor=true&cauthor_uid=18757996), [Erglis A](http://www.ncbi.nlm.nih.gov/pubmed/?term=Erglis%20A%5BAuthor%5D&cauthor=true&cauthor_uid=18757996), [Chechi T](http://www.ncbi.nlm.nih.gov/pubmed/?term=Chechi%20T%5BAuthor%5D&cauthor=true&cauthor_uid=18757996), [Ortolani P](http://www.ncbi.nlm.nih.gov/pubmed/?term=Ortolani%20P%5BAuthor%5D&cauthor=true&cauthor_uid=18757996), [Schalij MJ](http://www.ncbi.nlm.nih.gov/pubmed/?term=Schalij%20MJ%5BAuthor%5D&cauthor=true&cauthor_uid=18757996), [Diem P](http://www.ncbi.nlm.nih.gov/pubmed/?term=Diem%20P%5BAuthor%5D&cauthor=true&cauthor_uid=18757996), [Meier B](http://www.ncbi.nlm.nih.gov/pubmed/?term=Meier%20B%5BAuthor%5D&cauthor=true&cauthor_uid=18757996), [Windecker S](http://www.ncbi.nlm.nih.gov/pubmed/?term=Windecker%20S%5BAuthor%5D&cauthor=true&cauthor_uid=18757996), [Jüni P](http://www.ncbi.nlm.nih.gov/pubmed/?term=J%C3%BCni%20P%5BAuthor%5D&cauthor=true&cauthor_uid=18757996). Drug eluting and bare metal stents in people with and without diabetes: collaborative network meta-analysis. [BMJ.](http://www.ncbi.nlm.nih.gov/pubmed/18757996) 2008 Aug 29;337:a1331.

# [de Waha A](http://www.ncbi.nlm.nih.gov/pubmed/?term=de%20Waha%20A%5BAuthor%5D&cauthor=true&cauthor_uid=21221607)^1^, [Dibra A](http://www.ncbi.nlm.nih.gov/pubmed/?term=Dibra%20A%5BAuthor%5D&cauthor=true&cauthor_uid=21221607), [Kufner S](http://www.ncbi.nlm.nih.gov/pubmed/?term=Kufner%20S%5BAuthor%5D&cauthor=true&cauthor_uid=21221607), [Baumgart D](http://www.ncbi.nlm.nih.gov/pubmed/?term=Baumgart%20D%5BAuthor%5D&cauthor=true&cauthor_uid=21221607), [Sabate M](http://www.ncbi.nlm.nih.gov/pubmed/?term=Sabate%20M%5BAuthor%5D&cauthor=true&cauthor_uid=21221607), [Maresta A](http://www.ncbi.nlm.nih.gov/pubmed/?term=Maresta%20A%5BAuthor%5D&cauthor=true&cauthor_uid=21221607), [Schömig A](http://www.ncbi.nlm.nih.gov/pubmed/?term=Sch%C3%B6mig%20A%5BAuthor%5D&cauthor=true&cauthor_uid=21221607), [Kastrati A](http://www.ncbi.nlm.nih.gov/pubmed/?term=Kastrati%20A%5BAuthor%5D&cauthor=true&cauthor_uid=21221607). Long-term outcome after sirolimus-eluting stents versus bare metal stents in patients with diabetes mellitus: a patient-level meta-analysis of randomized trials. [Clin Res Cardiol.](http://www.ncbi.nlm.nih.gov/pubmed/21221607) 2011 Jul;100(7):561-70.

# [Qiao Y](http://www.ncbi.nlm.nih.gov/pubmed/?term=Qiao%20Y%5BAuthor%5D&cauthor=true&cauthor_uid=24217305)^1^, [Bian Y](http://www.ncbi.nlm.nih.gov/pubmed/?term=Bian%20Y%5BAuthor%5D&cauthor=true&cauthor_uid=24217305), [Yan X](http://www.ncbi.nlm.nih.gov/pubmed/?term=Yan%20X%5BAuthor%5D&cauthor=true&cauthor_uid=24217305), [Liu Z](http://www.ncbi.nlm.nih.gov/pubmed/?term=Liu%20Z%5BAuthor%5D&cauthor=true&cauthor_uid=24217305), [Chen Y](http://www.ncbi.nlm.nih.gov/pubmed/?term=Chen%20Y%5BAuthor%5D&cauthor=true&cauthor_uid=24217305). Efficacy and safety of sirolimus-eluting stents versus bare-metal stents in coronary artery disease patients with diabetes: a meta-analysis. [Cardiovasc J Afr.](http://www.ncbi.nlm.nih.gov/pubmed/24217305) 2013 Aug;24(7):274-9.

# [Boyden TF](http://www.ncbi.nlm.nih.gov/pubmed/?term=Boyden%20TF%5BAuthor%5D&cauthor=true&cauthor_uid=17493468)^1^, [Nallamothu BK](http://www.ncbi.nlm.nih.gov/pubmed/?term=Nallamothu%20BK%5BAuthor%5D&cauthor=true&cauthor_uid=17493468), [Moscucci M](http://www.ncbi.nlm.nih.gov/pubmed/?term=Moscucci%20M%5BAuthor%5D&cauthor=true&cauthor_uid=17493468), [Chan PS](http://www.ncbi.nlm.nih.gov/pubmed/?term=Chan%20PS%5BAuthor%5D&cauthor=true&cauthor_uid=17493468), [Grossman PM](http://www.ncbi.nlm.nih.gov/pubmed/?term=Grossman%20PM%5BAuthor%5D&cauthor=true&cauthor_uid=17493468), [Tsai TT](http://www.ncbi.nlm.nih.gov/pubmed/?term=Tsai%20TT%5BAuthor%5D&cauthor=true&cauthor_uid=17493468), [Chetcuti SJ](http://www.ncbi.nlm.nih.gov/pubmed/?term=Chetcuti%20SJ%5BAuthor%5D&cauthor=true&cauthor_uid=17493468), [Bates ER](http://www.ncbi.nlm.nih.gov/pubmed/?term=Bates%20ER%5BAuthor%5D&cauthor=true&cauthor_uid=17493468), [Gurm HS](http://www.ncbi.nlm.nih.gov/pubmed/?term=Gurm%20HS%5BAuthor%5D&cauthor=true&cauthor_uid=17493468). Meta-analysis of randomized trials of drug-eluting stents versus bare metal stents in patients with diabetes mellitus. [Am J Cardiol.](http://www.ncbi.nlm.nih.gov/pubmed/17493468) 2007 May 15;99(10):1399-402.

# [De Luca G](http://www.ncbi.nlm.nih.gov/pubmed/?term=De%20Luca%20G%5BAuthor%5D&cauthor=true&cauthor_uid=23490029)^1^, [Dirksen MT](http://www.ncbi.nlm.nih.gov/pubmed/?term=Dirksen%20MT%5BAuthor%5D&cauthor=true&cauthor_uid=23490029), [Spaulding C](http://www.ncbi.nlm.nih.gov/pubmed/?term=Spaulding%20C%5BAuthor%5D&cauthor=true&cauthor_uid=23490029), [Kelbæk H](http://www.ncbi.nlm.nih.gov/pubmed/?term=Kelb%C3%A6k%20H%5BAuthor%5D&cauthor=true&cauthor_uid=23490029), [Schalij M](http://www.ncbi.nlm.nih.gov/pubmed/?term=Schalij%20M%5BAuthor%5D&cauthor=true&cauthor_uid=23490029), [Thuesen L](http://www.ncbi.nlm.nih.gov/pubmed/?term=Thuesen%20L%5BAuthor%5D&cauthor=true&cauthor_uid=23490029), [van der Hoeven B](http://www.ncbi.nlm.nih.gov/pubmed/?term=van%20der%20Hoeven%20B%5BAuthor%5D&cauthor=true&cauthor_uid=23490029), [Vink MA](http://www.ncbi.nlm.nih.gov/pubmed/?term=Vink%20MA%5BAuthor%5D&cauthor=true&cauthor_uid=23490029), [Kaiser C](http://www.ncbi.nlm.nih.gov/pubmed/?term=Kaiser%20C%5BAuthor%5D&cauthor=true&cauthor_uid=23490029), [Musto C](http://www.ncbi.nlm.nih.gov/pubmed/?term=Musto%20C%5BAuthor%5D&cauthor=true&cauthor_uid=23490029), [Chechi T](http://www.ncbi.nlm.nih.gov/pubmed/?term=Chechi%20T%5BAuthor%5D&cauthor=true&cauthor_uid=23490029), [Spaziani G](http://www.ncbi.nlm.nih.gov/pubmed/?term=Spaziani%20G%5BAuthor%5D&cauthor=true&cauthor_uid=23490029), [Diaz de la Llera LS](http://www.ncbi.nlm.nih.gov/pubmed/?term=Diaz%20de%20la%20Llera%20LS%5BAuthor%5D&cauthor=true&cauthor_uid=23490029), [Pasceri V](http://www.ncbi.nlm.nih.gov/pubmed/?term=Pasceri%20V%5BAuthor%5D&cauthor=true&cauthor_uid=23490029), [Di Lorenzo E](http://www.ncbi.nlm.nih.gov/pubmed/?term=Di%20Lorenzo%20E%5BAuthor%5D&cauthor=true&cauthor_uid=23490029), [Violini R](http://www.ncbi.nlm.nih.gov/pubmed/?term=Violini%20R%5BAuthor%5D&cauthor=true&cauthor_uid=23490029), [Suryapranata H](http://www.ncbi.nlm.nih.gov/pubmed/?term=Suryapranata%20H%5BAuthor%5D&cauthor=true&cauthor_uid=23490029), [Stone GW](http://www.ncbi.nlm.nih.gov/pubmed/?term=Stone%20GW%5BAuthor%5D&cauthor=true&cauthor_uid=23490029); [DESERT Cooperation](http://www.ncbi.nlm.nih.gov/pubmed/?term=DESERT%20Cooperation%5BCorporate%20Author%5D). Meta-analysis comparing efficacy and safety of first generation drug-eluting stents to bare-metal stents in patients with diabetes mellitus undergoing primary percutaneous coronary intervention. [Am J Cardiol.](http://www.ncbi.nlm.nih.gov/pubmed/23490029) 2013 May 1;111(9):1295-304.

# [Pan XH](http://www.ncbi.nlm.nih.gov/pubmed/?term=Pan%20XH%5BAuthor%5D&cauthor=true&cauthor_uid=20872982)^1^, [Chen YX](http://www.ncbi.nlm.nih.gov/pubmed/?term=Chen%20YX%5BAuthor%5D&cauthor=true&cauthor_uid=20872982), [Xiang MX](http://www.ncbi.nlm.nih.gov/pubmed/?term=Xiang%20MX%5BAuthor%5D&cauthor=true&cauthor_uid=20872982), [Xu G](http://www.ncbi.nlm.nih.gov/pubmed/?term=Xu%20G%5BAuthor%5D&cauthor=true&cauthor_uid=20872982), [Wang JA](http://www.ncbi.nlm.nih.gov/pubmed/?term=Wang%20JA%5BAuthor%5D&cauthor=true&cauthor_uid=20872982). A meta-analysis of randomized trials on clinical outcomes of paclitaxel-eluting stents versus bare-metal stents in ST-segment elevation myocardial infarction patients. [J Zhejiang Univ Sci B.](http://www.ncbi.nlm.nih.gov/pubmed/20872982) 2010 Oct;11(10):754-61.

# [Patti G](http://www.ncbi.nlm.nih.gov/pubmed/?term=Patti%20G%5BAuthor%5D&cauthor=true&cauthor_uid=18993150), [Nusca A](http://www.ncbi.nlm.nih.gov/pubmed/?term=Nusca%20A%5BAuthor%5D&cauthor=true&cauthor_uid=18993150), [Di Sciascio G](http://www.ncbi.nlm.nih.gov/pubmed/?term=Di%20Sciascio%20G%5BAuthor%5D&cauthor=true&cauthor_uid=18993150). Meta-analysis comparison (nine trials) of outcomes with drug eluting stents versus bare metal stents in patientswith diabetes mellitus. [Am J Cardiol.](http://www.ncbi.nlm.nih.gov/pubmed/?term=Meta-analysis+comparison+(nine+trials)+of+outcomes+with+drug-eluting+stents+versus+bare+metal+stents+in+patients+with+diabetes+mellitus) 2008 Nov 15;102(10):1328-34.

# [Kastrati A](http://www.ncbi.nlm.nih.gov/pubmed/?term=Kastrati%20A%5BAuthor%5D&cauthor=true&cauthor_uid=17901079)^1^, [Dibra A](http://www.ncbi.nlm.nih.gov/pubmed/?term=Dibra%20A%5BAuthor%5D&cauthor=true&cauthor_uid=17901079), [Spaulding C](http://www.ncbi.nlm.nih.gov/pubmed/?term=Spaulding%20C%5BAuthor%5D&cauthor=true&cauthor_uid=17901079), [Laarman GJ](http://www.ncbi.nlm.nih.gov/pubmed/?term=Laarman%20GJ%5BAuthor%5D&cauthor=true&cauthor_uid=17901079), [Menichelli M](http://www.ncbi.nlm.nih.gov/pubmed/?term=Menichelli%20M%5BAuthor%5D&cauthor=true&cauthor_uid=17901079), [Valgimigli M](http://www.ncbi.nlm.nih.gov/pubmed/?term=Valgimigli%20M%5BAuthor%5D&cauthor=true&cauthor_uid=17901079), [Di Lorenzo E](http://www.ncbi.nlm.nih.gov/pubmed/?term=Di%20Lorenzo%20E%5BAuthor%5D&cauthor=true&cauthor_uid=17901079), [Kaiser C](http://www.ncbi.nlm.nih.gov/pubmed/?term=Kaiser%20C%5BAuthor%5D&cauthor=true&cauthor_uid=17901079), [Tierala I](http://www.ncbi.nlm.nih.gov/pubmed/?term=Tierala%20I%5BAuthor%5D&cauthor=true&cauthor_uid=17901079), [Mehilli J](http://www.ncbi.nlm.nih.gov/pubmed/?term=Mehilli%20J%5BAuthor%5D&cauthor=true&cauthor_uid=17901079), [Seyfarth M](http://www.ncbi.nlm.nih.gov/pubmed/?term=Seyfarth%20M%5BAuthor%5D&cauthor=true&cauthor_uid=17901079), [Varenne O](http://www.ncbi.nlm.nih.gov/pubmed/?term=Varenne%20O%5BAuthor%5D&cauthor=true&cauthor_uid=17901079), [Dirksen MT](http://www.ncbi.nlm.nih.gov/pubmed/?term=Dirksen%20MT%5BAuthor%5D&cauthor=true&cauthor_uid=17901079),[Percoco G](http://www.ncbi.nlm.nih.gov/pubmed/?term=Percoco%20G%5BAuthor%5D&cauthor=true&cauthor_uid=17901079), [Varricchio A](http://www.ncbi.nlm.nih.gov/pubmed/?term=Varricchio%20A%5BAuthor%5D&cauthor=true&cauthor_uid=17901079), [Pittl U](http://www.ncbi.nlm.nih.gov/pubmed/?term=Pittl%20U%5BAuthor%5D&cauthor=true&cauthor_uid=17901079), [Syvänne M](http://www.ncbi.nlm.nih.gov/pubmed/?term=Syv%C3%A4nne%20M%5BAuthor%5D&cauthor=true&cauthor_uid=17901079), [Suttorp MJ](http://www.ncbi.nlm.nih.gov/pubmed/?term=Suttorp%20MJ%5BAuthor%5D&cauthor=true&cauthor_uid=17901079), [Violini R](http://www.ncbi.nlm.nih.gov/pubmed/?term=Violini%20R%5BAuthor%5D&cauthor=true&cauthor_uid=17901079), [Schömig A](http://www.ncbi.nlm.nih.gov/pubmed/?term=Sch%C3%B6mig%20A%5BAuthor%5D&cauthor=true&cauthor_uid=17901079). Meta-analysis of randomized trials on drug-eluting stents vs. bare-metal stents in patients with acute myocardial infarction. [Eur Heart J.](http://www.ncbi.nlm.nih.gov/pubmed/17901079) 2007 Nov;28(22):2706-13.

# [Hao PP](http://www.ncbi.nlm.nih.gov/pubmed/?term=Hao%20PP%5BAuthor%5D&cauthor=true&cauthor_uid=20978561)^1^, [Chen YG](http://www.ncbi.nlm.nih.gov/pubmed/?term=Chen%20YG%5BAuthor%5D&cauthor=true&cauthor_uid=20978561), [Wang XL](http://www.ncbi.nlm.nih.gov/pubmed/?term=Wang%20XL%5BAuthor%5D&cauthor=true&cauthor_uid=20978561), [Zhang Y](http://www.ncbi.nlm.nih.gov/pubmed/?term=Zhang%20Y%5BAuthor%5D&cauthor=true&cauthor_uid=20978561). Efficacy and safety of drug-eluting stents in patients with acute ST-segment-elevation myocardial infarction: a meta-analysis of randomized controlled trials. [Tex Heart Inst J.](http://www.ncbi.nlm.nih.gov/pubmed/20978561) 2010;37(5):516-24.

# [Geng DF](http://www.ncbi.nlm.nih.gov/pubmed/?term=Geng%20DF%5BAuthor%5D&cauthor=true&cauthor_uid=22899538)^1^, [Meng Z](http://www.ncbi.nlm.nih.gov/pubmed/?term=Meng%20Z%5BAuthor%5D&cauthor=true&cauthor_uid=22899538), [Yan HY](http://www.ncbi.nlm.nih.gov/pubmed/?term=Yan%20HY%5BAuthor%5D&cauthor=true&cauthor_uid=22899538), [Nie RQ](http://www.ncbi.nlm.nih.gov/pubmed/?term=Nie%20RQ%5BAuthor%5D&cauthor=true&cauthor_uid=22899538), [Deng J](http://www.ncbi.nlm.nih.gov/pubmed/?term=Deng%20J%5BAuthor%5D&cauthor=true&cauthor_uid=22899538), [Wang JF](http://www.ncbi.nlm.nih.gov/pubmed/?term=Wang%20JF%5BAuthor%5D&cauthor=true&cauthor_uid=22899538). Bare-metal stent versus drug-eluting stent in large coronary arteries: meta-analysis of randomized controlled trials. [Catheter Cardiovasc Interv.](http://www.ncbi.nlm.nih.gov/pubmed/22899538) 2013 Jun 1;81(7):1087-94.
