# Supplementary material for: Comparing the Clinical Outcomes between Drug Eluting Stents and Bare Metal Stents in Patients with Insulin-Treated Type 2 Diabetes Mellitus: A Systematic Review and Meta-Analysis of 10 Randomized Controlled Trials
Source: PLoS One. 2016 Apr 25;11(4):e0154064. doi: 10.1371/journal.pone.0154064 (PMC4844102; doi:10.1371/journal.pone.0154064)
Supplement: S1 Flow Diagram — (DOC) [file pone.0154064.s003.doc]

Records identified through Medline and Embase
(n = 679)

Records after duplicates removed
(n =592)

Records screened
(n = 592)

Records excluded since not related to our topic
(n = 535)

Full-text articles assessed for eligibility
(n = 57)

Full-text articles excluded because they were:

1. Non.RCTs, Meta-analysis and case studies (n=35).
2. Data for insulin-treated DM patients could not be extracted (n= 12)

RCTs included in this meta-analysis
(n = 10)
